# Contemporary exploitation of natural products for arthropod-borne pathogen transmission-blocking interventions

**DOI:** 10.1186/s13071-022-05367-8

**Published:** 2022-08-24

**Authors:** Jackson M. Muema, Joel L. Bargul, Meshack A. Obonyo, Sospeter N. Njeru, Damaris Matoke-Muhia, James M. Mutunga

**Affiliations:** 1 Department of Biochemistry, Jomo Kenyatta University of Agriculture and Technology (JKUAT), P.O. Box 62000, Nairobi, 00200 Kenya; 2 International Centre of Insect Physiology and Ecology (Icipe), P.O. Box 30772, Nairobi, 00100 Kenya; 3 Department of Biochemistry and Molecular Biology, Egerton University, P.O. Box 536, Egerton, 20115 Kenya; 4 Centre for Traditional Medicine and Drug Research (CTMDR), Kenya Medical Research Institute (KEMRI), P.O. Box 54840, Nairobi, 00200 Kenya; 5 Centre for Biotechnology Research Development (CBRD), Kenya Medical Research Institute (KEMRI), P.O. Box 54840, Nairobi, 00200 Kenya; 6 Department of Biological Sciences, Mount Kenya University (MKU), P.O. Box 54, Thika, 01000 Kenya; 7 Present Address: School of Engineering Design, Technology and Professional Programs, Pennsylvania State University, University Park, PA 16802 USA

**Keywords:** Human pathogen transmission-blocking, Natural products, Arthropod disease vectors, Disease control, Anti-infectives

## Abstract

An integrated approach to innovatively counter the transmission of various arthropod-borne diseases to humans would benefit from strategies that sustainably limit onward passage of infective life cycle stages of pathogens and parasites to the insect vectors and vice versa. Aiming to accelerate the impetus towards a disease-free world amid the challenges posed by climate change, discovery, mindful exploitation and integration of active natural products in design of pathogen transmission-blocking interventions is of high priority. Herein, we provide a review of natural compounds endowed with blockade potential against transmissible forms of human pathogens reported in the last 2 decades from 2000 to 2021. Finally, we propose various translational strategies that can exploit these pathogen transmission-blocking natural products into design of novel and sustainable disease control interventions. In summary, tapping these compounds will potentially aid in integrated combat mission to reduce disease transmission trends.

**Supplementary Information:**

The online version contains supplementary material available at 10.1186/s13071-022-05367-8.

## Background

Haematophagous arthropod vectors—mainly mosquitoes, phlebotomine sand flies, triatomine bugs, simulid blackflies and tsetse flies—inadvertently transmit highly infectious pathogens to humans during blood meal acquisition. Arboviruses (chikungunya virus, CHIKV; Zika virus, ZIKV; dengue fever virus, DENV; West Nile virus, WNV; Rift Valley fever virus, RVFV; sand fly fevers, yellow fever virus, YFV, etc.), lymphatic filarial worms, *Wuchereria* *bancrofti*, *Brugia* spp*.*, *Plasmodium* parasites, *Onchocerca* *volvulus* and kinetoplastids (leishmania and trypanosomes) that develop in these insects gravely afflict humans residing in tropical and subtropical regions. These vector-borne pathogens contribute to > 17% of all human infectious diseases, accounting for > 700,000 annual deaths estimated by the World Health Organization (WHO) (https://www.who.int/news-room/fact-sheets/detail/vector-borne-diseases). The successful transmission of these pathogens across vertebrate and insect hosts is facilitated by intricate multi-ecological factors, most of which remain poorly understood, yet intriguingly providing excellent opportunities for novel intervention designs. While the development of effective vaccines against these arthropod-borne diseases appears far beyond reach, translational aspects to counter the infectious bites especially through small molecule forms are warranted.

The attractiveness to infectious individuals in some insect vectors for pathogen uptake is the initial adaptive success towards sustained transmission. For instance, in malaria highly gametocytemic individuals have modulated host skin microbiota composition and chemistry that generates volatile organic compounds (VOCs), which attract more female *Anopheles* mosquitoes for blood feedings, and in the process acquire *Plasmodium* parasites [1–4]. A similar phenomenon has been reported for sand flies [5]. Such host-induced attractiveness is however lacking for many emerging and re-emerging arboviruses partly because these mosquito infections are incidental, but also other evolved viral mechanisms have been described to sustain their transmission cycle upon viral establishment [6]. These viral mechanisms include vertical (transovarial and trans-egg transmissions) and horizontal transmission, or both. However, other viruses find their way into mosquitoes through environmental acquisition; for instance, ZIKVs have been reported to potentially infect mosquito juveniles while breeding in contaminated sites [7]. Nevertheless, following the ingestion of infectious blood meals the developing pathogens must infect various tissues and evade physiological bottlenecks imposed by the vector immune defences to facilitate successful transformation into infective stages ready for transmission to the next human host. This infective passage from the vector to humans occurs during appetite-induced search for blood meal, but also as a result of enhanced manipulative feeding effects by the infective stages as in the case of malaria, leishmania and DENV [8]. A consequential worrying trend is the possible transmission of drug-resistant pathogens, exemplified by *Plasmodium* sporozoites, CHIKV variants and leishmania promastigotes that have been reported [9–12].

With current global elimination strategies for infectious diseases in focus, there is a dire need to find appropriate transmission-reducing interventions [13–16]. In fact, the UN Sustainable Development Goal 3 advocates for elimination of human infectious pathogens by 2030 anchored on target 3.3. Towards this goal, knowledge on transmission of insect-vectored human pathogens between host interfaces continues to grow rapidly in tandem with recent advancements of -omics technologies and potential drug targets discovered. Prioritizations have largely taken the form of disrupting either the development of transmissible stages in human and insect hosts or the activation factors of quiescent infectious forms, as well as targeting the host–pathogen interaction proteins that offer less drug pressure in the insect vector [17]. Opportunities, viz, (i) arbitrary screening for direct cidal activity against transmissible stages, (ii) targeting the host factors functionally identified to facilitate host cell invasion, replication and egression, (iii) exploitation of obligate endosymbionts, (iv) vector sugar feeding behaviour and endectocides, (v) enzyme inhibitions of essential pathogen proteins and (vii) *Plasmodium* liver stages following sporozoite inoculation have taken centre stage in the identification of transmission-blocking compounds [18–23].

**Fig. 1:**
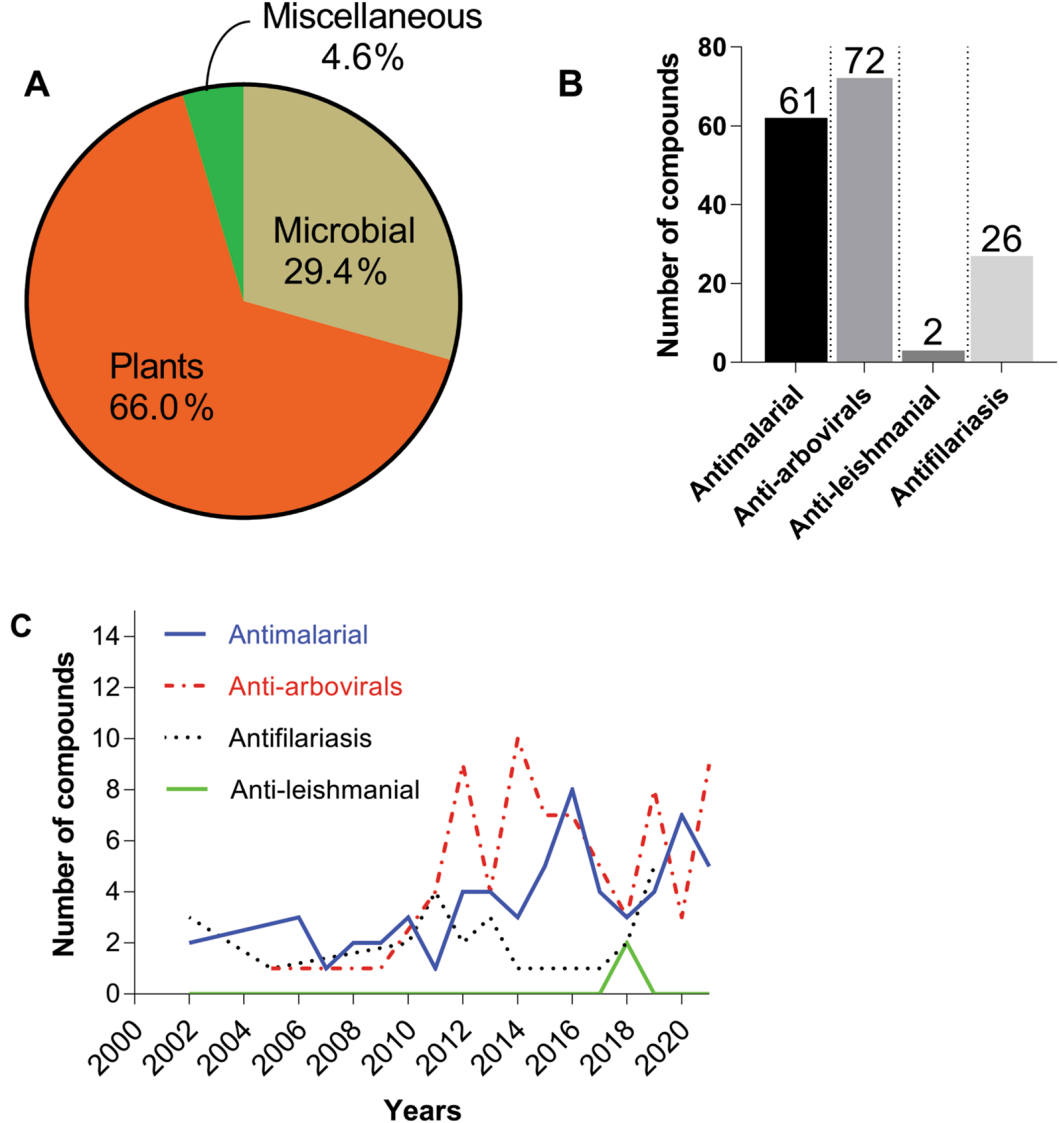
Summarized analyses of the highlighted compounds retrieved from literature.** A** Overall distribution of sources of natural compounds highlighted in this review. **B** Disease target profile of the highlighted natural compounds. **C** Trends in exploration of natural products in pursuit of pathogen transmission-blocking between 2000 and 2021

Despite a slow pace in their exploration, compared to chemotherapeutic counterparts, natural products (primarily from plants, microbial sources, marine organisms, insect microbiomes and higher invertebrates) and their synthetic derivatives as pathogen transmission-blocking agents could offer an integral pivot in disease control and prevention. Structural diversity and complexity provided by these compounds have been widely appreciated in drug discovery as novel lead scaffolds for various anti-infective drugs [24, 25]. Within this context, the identification and strategic use of natural products as new leads to combat further spread of vector-borne diseases to humans will complement the existing interventions. Our earlier studies have contributed to these efforts by documenting the vector control strategies utilizing natural compounds [26]. In this review, we provide a summary based on an extensive literature search on the promising anti-infective natural compounds (and their derivatives) discovered in the last 22 years (from 2000 to 2021). The scientific literature was searched from PubMed, Google Scholar, Wiley Online Library, ScienceDirect, ACS Publications, Royal Society of Chemistry (RSC), Web of Science and SpringerLink libraries. Relevant keywords, “transmission-blocking”, “natural products”, “human vector diseases”, “antivirals”, “procyclic and metacyclic trypomastigotes”, “arboviruses”, “antifilariasis”, “anti-wolbachial”, and “procyclic promastigotes” and appropriate combinations of the above terms, were used to retrieve the articles. Our analyses demonstrate a great emphasis on exploration of plant- (66.01%) and microbial-derived (29.41%) chemical space in pursuit of reducing the spread of malaria (61 compounds), arboviruses (72 compounds), lymphatic filariasis (26 compounds) and leishmania (2 compounds) transmissions over the 22-year period (Fig. 1A–C). Moreover, we present our perspectives on various prospective applicability strategies of these molecules towards impedance of transmission of infectious pathogens between humans and insect vectors.

## Transmission-blocking in pursuit of human disease elimination and eradication agenda: the challenge meets nature?

### Midgut microbiota and microbial explorations

**Fig. 2:**
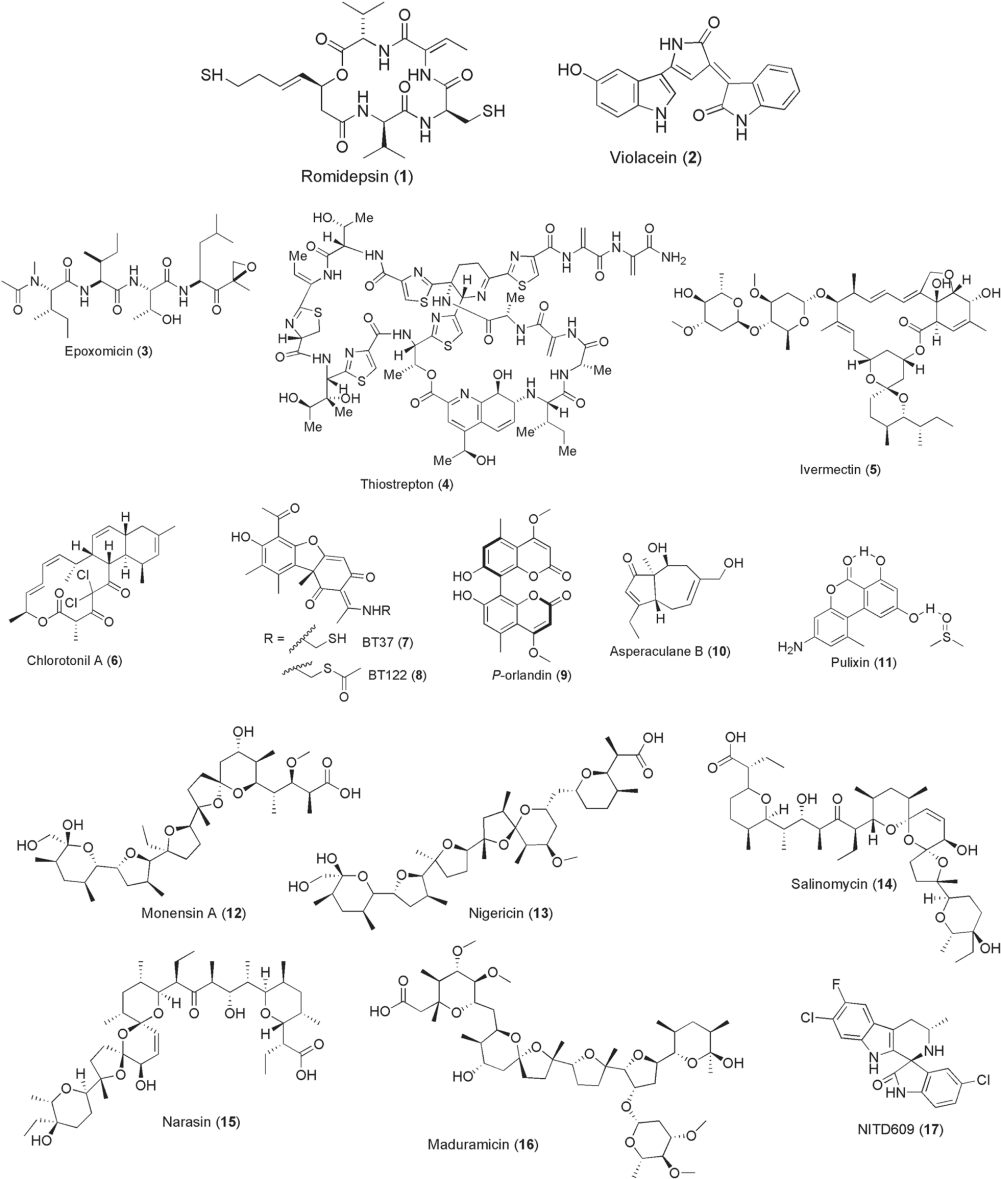
Chemical structures of compounds **1**–**17**. Romidepsin (**1**), Violacein (**2**), Epoxomicin (**3**), thiostrepton (**4**), ivermectin (**5**), chlorotonil a (**6**), bt37 (**7**), bt122 (**8**), p-orlandin (**9**), asperaculane b (**10**), pulixin (**11**), monensin a (**12**), nigericin (**13**), salinomycin (**14**), narasin (**15**), maduramicin (**16**), NITD609 (**17**)

Naturally most if not all living organisms, including disease-transmitting insect vectors, are colonized by multigenera microbial communities existing in unresolved complex symbiotic interactions largely shaped by their proximate environment [27]. Wild-caught mosquitoes, for instance, are dominated by highly diverse and dynamic gram-negative bacteria, *Pseudomonas*, *Aeromonas*, *Asaia*, *Comamonas*, *Elizabethkingia*, *Serratia*, *Acinetobacter*, *Enterobacter*, *Klebsiella* and *Pantoea*, and symbiotic fungi; yeast, *Candida*, *Penicillium*, and *Pichia* (reviewed in [28]). Antiparasitic, antimicrobial and antiviral products secreted by such microbes (inclusive of insect-associated symbionts) are or have found interesting pharmaceutical applications [29–31]. Several studies published over the recent years have demonstrated the potential impact of gut bacterial products in impairing *Plasmodium* sporogonic development through direct killing, indirect immunomodulatory effects, or both. *Chromobacterium* spp. (*Enterobacter* bacterium) isolated from midguts of field-caught Zambian anophelines (*Esp*_Z) [32] and Panamanian *Aedes* *aegypti* mosquitoes (*Csp_*P) [33] inhibited *Plasmodium* ookinete development and also abrogated replication of dengue virus in mosquitoes. These inhibitory activities were later found to be mediated by a histone deacetylase inhibitor romidepsin (**1**; Fig. 2) and aminopeptidase secretion, respectively [34, 35]. The depsipeptide compound **1** has potent *Plasmodium* gametocytocidal activities. Besides, the bacterial isolate (*Csp*_P) kills mosquito larvae by its secreted hydrogen cyanide in addition to exerting adulticidal effects [36], underscoring its broad-spectrum activity window. A related *Chromobacterium* species *C.* *violaceum* has been isolated from soils [37]. These bacteria produce a broad-spectrum bisindole antimalarial agent, 3-[1,2-dihydro-5-(5-hydroxy-1*H*-indol-3-yl)-2-oxo-3*H*-pyrrol-3-ilydeno]-1,3-dihydro-2*H*-indol-2-one (violacein (**2**)), reported to have gametocytocidal (EC_50_ 1.25–2.5 µM) and *Plasmodium* transmission-blocking activity (43% oocysts reduction) [37]. In another more recent study, despite pending efforts to establish the identity of anti-*Plasmodium* mediating molecule(s), a novel sexually inherited *microsporidian* MB infecting Kenyan *An*. *arabiensis* population was discovered to block transmission of *P.* *falciparum* (undetectable levels of sporozoites) [38, 39]. Similar bacteria with *P.* *vivax* transmission-blocking activity are the *Serratia* spp. and *Enterobacter* spp. isolated from midguts of field-collected *An*. *albimanus* in southern Mexico, affording appreciable reductions in oocysts densities [40]. Further exploration of these bacterial species, and their bioactive compounds **1**, **2** as potential leishmania, *Trypanosoma*, filarial and arboviral transmission-blocking interventions, is lacking in literature and presents an open path of scientific exploration. Transmission-blocking endosymbiotic “killer” yeast strains of *Wickerhamomyces* *anomalus* have been shown to undergo maternal inheritance in different insects, including *Anopheles*, *Aedes*, and *Culex* mosquitoes, and sand flies [41–43]. Secreted antimicrobial toxins such as *Wa*F17.12 and *Wa*ATCC96603 induced in vitro killing of *P.* *berghei* ANKA sporogonic stages at LC_50_ 64.6 µg/ml and 61.3 µg/ml, respectively, reducing 65.2% of early sporogonic parasites and oocysts in mosquitoes [44, 45]. Stable pathogen transmission-blocking compounds from symbionts in other disease vectors are yet to be identified.

Equally important are the microbial products from ubiquitous bacteria and fungi. Exploitation of such microbial-derived compounds as human pathogen transmission-blockers is widely documented, but largely limited to malaria, filariasis, and arboviruses. Early in 2009, epoxomicin (**3**), a known proteosome degradation inhibitor discovered from an Actinomycetes strain Q996-17, emerged to potently kill mature *Plasmodium* stage V gametocytes at 100 or 10 nM 72-h post-treatment and blocked oocysts production in mosquitoes [46]. Subsequent work on plasmodial proteosome led to the identification of a *Streptomycetes* spp.-derived thiopeptide, thiostrepton (**4**), and its semisynthetic derivatives SS231/[14] and SS234/[05] with promising gametocytocidal activities in the IC_50_ range of 1.82–3.40 µM [47]. From *Streptomyces* *avermectinius* bacterium identified back in early 1970s by a Japanese microbiologist, Satoshi Omura, a macrocyclic lactone ivermectin (**5**) was pioneered and developed in 1983 by a Merck’s team led by William Campbell as an anthelmintic drug [48]. A veterinary drug trademarked as Mectizan^®^ was later prioritized for mass drug administration (MDA) at an oral dose of 150–200 µg/kg to treat river blindness prevalent in West Africa, Yemen, and Latin America regions. Apart from this remarkable invention, compound **5** has been repurposed successfully in malaria exerting endectocidal, *Plasmodium* sporontocidal, and sporogonic inhibitions at both laboratory and field levels [49–52]. The systemic administration and/or topical application of compound **5** to cattle and humans reduces the survival, feeding frequencies, blood digestibility, locomotion, and fecundity of bloodsucking arthropod disease vectors including mosquitoes and tsetse flies [53–57]. As human malaria transmission greatly relies on the longevity of female mosquitoes, reduction of their survivorship rates from this intervention breaks parasite transmission especially by the outdoor feeding vectors.

Another tricyclic macrolide, chlorotonil A (**6**), that structurally resembles the antibiotic anthracimycin was discovered from a soil-dwelling myxobacterium *Sorangium* *cellulosum* ce1525 in Germany. Chlorotonil A exerts nanomolar potency against late-stage IV/V gametocytes (IC_50_ 29.6 nM; IC_90_ 123.2 nM), besides its antimalarial activity against all intraerythrocytic stages [58]. This notable bioactivity has however not been extended for investigations against either the *Plasmodium* sporogonic stages or repurposed to target other arthropod transmissible human pathogens. Furthermore, lichen-derived (+)-usnic acid derivatives of dibenzofurandione class, BT37 (**7**) and BT122 (**8**), prevent *Plasmodium* zygote-to-ookinete maturation achieving 100% inhibition of oocyst production at 250 µg/ml [59]. (+)-Usnic acid also inhibits *P.* *berghei* liver stages at IC_50_ value of 2.3 µM, but is less active against *P.* *falciparum* blood stages [60]. It has also been demonstrated that fibrinogen-related proteins (FREPs) from *An*. *gambiae* midgut epithelium, and specifically FREP1, facilitate *Plasmodium* ookinete invasion through surface anchorage [61]. They observed that silencing of FREP1 gene expression reduces *P.* *falciparum* infection in mosquitoes. Inspired by these findings, through a systematic screening of the fungal library by ELISA-based method, a bicoumarin p-orlandin (**9**) from *Aspergillus* *niger* emerged as a potential candidate that prevents either *Plasmodium* gametocytes or ookinetes from interacting with FREP1 at 92% inhibition [62]. Consequently, such disruption, as at low dose of 3 µg/ml, effectively reduced *P.* *falciparum* infection load in mosquitoes by 56.7% oocyst numbers and 35% infection prevalence. In continuation with this work by Jun Li’s group, other fungal compounds, Asperaculane B (**10**) and [3-amino-7,9-dihydroxy-1-methyl-6*H*-benzo[c]chromen-6-one (Pulixin, (**11**)], with *Plasmodium* transmission-blocking activities have been identified [63, 64]. Compound **10** derived from *Aspergillus* *aculeatus* inhibits *Plasmodium* transmission at IC_50_ 7.89 µM, while **11** isolated from *Purpureocillium* *lilacinum* exerts its activity in mosquitoes at EC_50_ 11 µM, without notable host cytotoxicity.

A chemical class of polyether ionophores comprising monensin A (**12**), nigericin (**13**), salinomycin (**14**), narasin (**15**), and maduramicin (**16**), to mention a few, is reportedly derived from Actinobacteria of *Streptomyces* spp. These specific lipid-soluble monovalent compounds tend to bind metal cations reversibly at high affinities, transporting them across the cell membrane and disrupting parasite intracellular ionic homeostasis. Apart from exhibiting nanomolar activity against asexual stages of *Plasmodium*, the repurposed ionophores **12–16** preferentially kill transmissible stage IV/V gametocytes and liver stages more rapidly and impair sporogonic development in mosquitoes at nanomolar doses [65–69]. Such potent susceptibility of gametocytes, in addition to parasite transmission-blocking activity, to ionic balance perturbation is akin to PfATP4 targeting by spiroindolone NITD609 (**17**) [70, 71]. NITD609 (also referred to as cipargamin or KAE609) is a clinical trial phase 2 candidate discovered and developed from a screen of ~ 12,000 Novartis microbial product library.

**Fig. 3:**
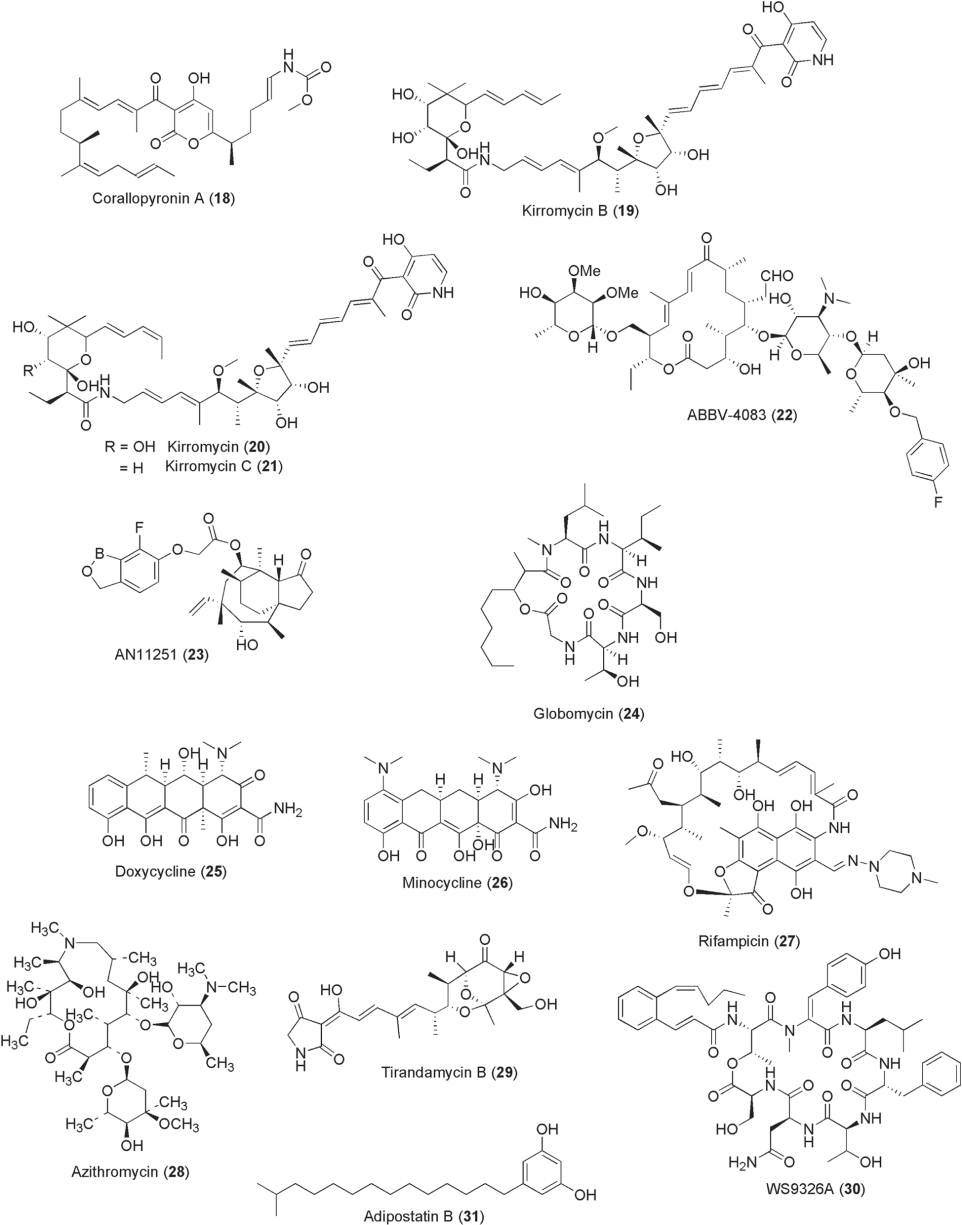
Chemical structures of compounds **18**–**31**. Corallopyronin A (CorA) (**18**), kirromycin b (**19**), kirromycin (**20**), kirromycin c (**21**), abbv-4083 (**22**), an11251 (**23**), globomycin (**24**), doxycycline (**25**), minocycline (**26**), rifampicin (**27**), azithromycin (**28**), tirandamycin b (**29**), WS9326a (**30**), adipostatin compound (**31**)

For lymphatic filariasis, efforts to discover new treatment options with transmission-blocking capability to complement ivermectin (**5**) are to date targeting the obligate filarial endosymbiotic bacterium *Wolbachia* by utilizing suitable platforms of insect cell lines. Chemical depletion of *Wolbachia* from filarial worms renders them infertile and nonviable, blocks embryogenesis, and inhibits their development, potentially blocking parasite transmission. In this regard, adult worm-sterilizing compounds such as corallopyronin A (CorA) (**18**; Fig. 3) from *Corallococcus* *coralloides* B035 that specifically depletes *Wolbachia* from filarial nematodes are showing promising preclinical candidature [72, 73]. CorA effectively targets bacterial RNA polymerase. When offered to infected *Litomosoides* *sigmodontis* rodent model for 14 days, > 90% of *Wolbachia* from filarial worms were cleared and development of adult worms abrogated, exerting a short-course efficacy in combination with albendazole for only 7 days at 10 mg/kg CorA [72]. Elsewhere, Xu et al. [74] discovered kirromycins–kirromycin B (**19**) and congeners kirromycin (**20**) and kirromycin C (**21**) from *Streptomyces* sp. CB00686 through a high-throughput screening of a natural product library consisting of 348 compounds isolated from 65 bacterial strains at The Scripps Research Institute. The three kirromycins **19**–**21** potently depleted *Wolbachia* in LDW1 *Drosophila* cells (IC_50_ 0.58, 0.25, 1.08 nM, respectively) and *Brugia* *pahangi* ovaries ex vivo (65–95% efficiency at 1 µM). Such anti-*Wolbachia* activity of **19**–**21** is believed to originate from inhibition of protein synthesis through interaction with prokaryotic elongation factor Tu (EF-Tu). From a discovered macrolide tylosin A (TylA) of *Streptomyces* *fradiae*, von Geldern et al. [75] esterified the 4″-OH on mycarose sugar to develop ABBV-4083 (**22**) with potent anti-*Wolbachia* activity (EC_50_ 0.019 nM), in vivo efficacy of > 99.9% at 150 mg/kg for 14 days, and superior pharmacokinetic profile. Another modified antibiotic boron-pleuromutilin, AN11251 (**23**), exerts enhanced in vitro anti-*Wolbachia* activity of EC_50_ 15 nM compared to less active pleuromutilin itself (EC_50_ > 1 µM) [76]. Oral administration of compound **23** at 50 mg/kg to *L.* *sigmodontis* mouse infection model for 14 days effectively clears > 99% of *Wolbachia* from adult female worms. Globomycin (**24**) is yet another filaricidal agent, first isolated in 1978 from fermentation of *Streptomyces* spp. [77]. Globomycin is known for its lipoprotein signal peptidase II (LspA) inhibitory activity. By inhibiting lipoprotein biosynthesis, globomycin depletes *Wolbachia* from *B.* *malayi* and kills the adult worms at 100 µg/ml [78].

Other compounds with anti-*Wolbachia* and antifilarial activity are the antibiotics doxycycline (**25**; Fig. 3), minocycline (**26**): two synthetic derivatives of *Streptomyces*-derived tetracycline, rifampicin (**27**): a product of a soil bacterium *Amycolatopsis* *rifamycinica*, and azithromycin (**28**): a semisynthetic macrolide derivative of *Streptomyces* derived erythromycin [79]. In a recent study report by Bullman et al. [80], however, the antibiotics **25**–**27** displayed inverse treatment effects on *Wolbachia,* increasing titres with dose increment, despite rendering *Brugia* worms moribund. Besides these advances, microbial compounds of *Streptomyces* spp. origin targeting asparaginyl-tRNA synthetase activity are able to kill adult *Brugia* worms. These include tirandamycin B (**29**), WS9326A (**30**), and adipostatins A-D [structurally represented by (**31**)] identified via high throughput screening [81–83]. Notably, the aforementioned compounds **18**–**31** have only been tested in vitro and using mouse infection models, raising a concern as to whether the observed activity is also extrapolatable to insect vector level from an infectious human blood meal. This arises owing to a compendium of interactive factors influencing transmissibility of human pathogens that are potentially missed by in vitro assay conditions.

**Fig. 4:**
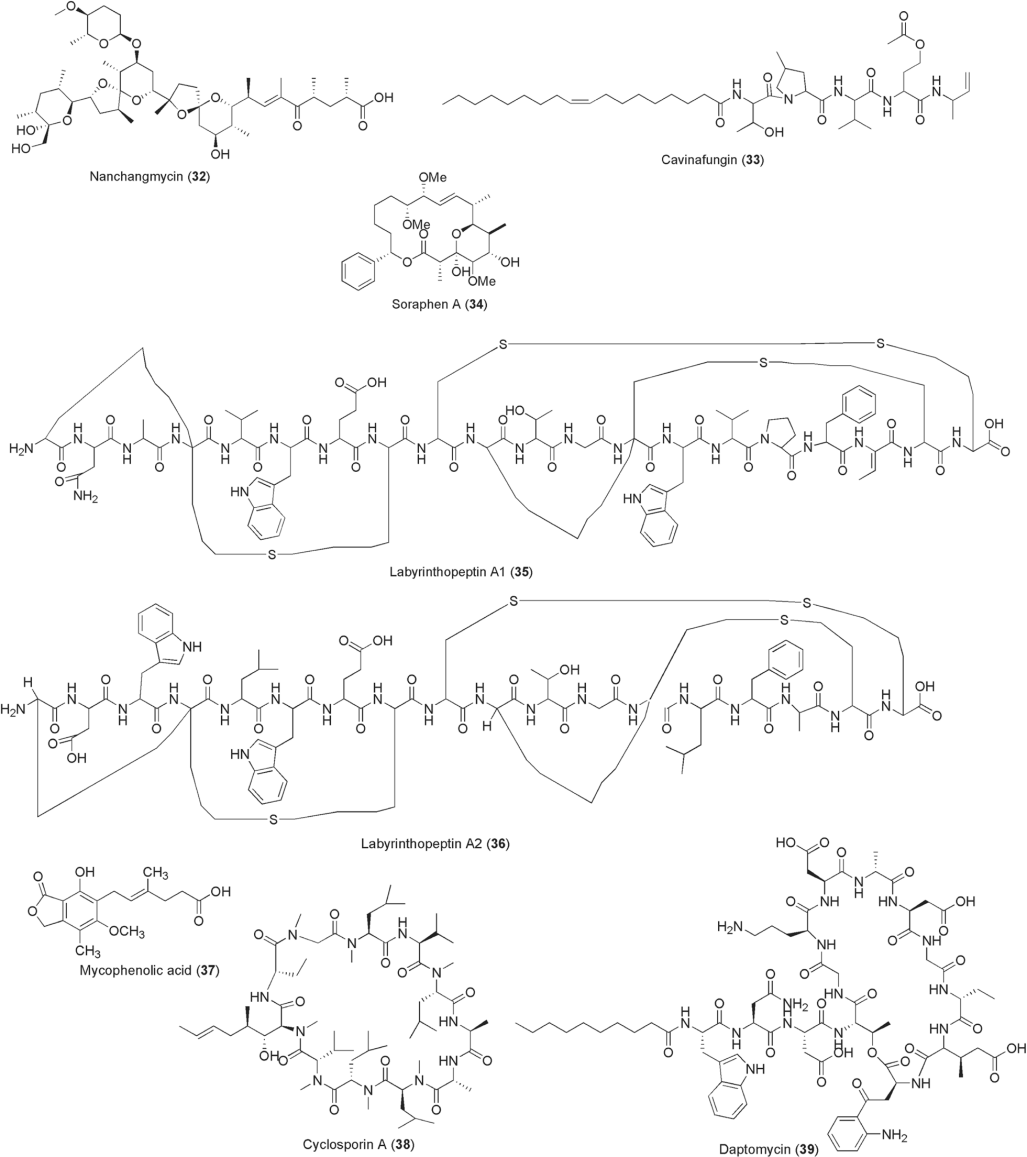
Chemical structures of compounds **32**–**39**. Nanchangmycin (**32**), cavinafungin (**33**), soraphen a (**34**), labyrinthopeptins a1 (**35**), labyrinthopeptins a2 (**36**), mycophenolic acid (mpa) (**37**), cyclosporine a (**38**), daptomycin (**39**)

Reduction of arboviral titres at the points of acquisition and inoculation through chemical inhibition of host factors required for viral replication is a promising approach for blocking transmissions of arboviruses. However, only a few examples of comparable inhibitors of microbial origin are known at present and they are highlighted below. Nanchangmycin [**32**; isolated from *Streptomyces* *nanchangensis* (Fig. 4)] was identified through screening a 2000 compound library and established to block ZIKV infection at EC_50_ 0.97 µM by targeting the human viral attachment factor AXL [84]. In addition, this ionophore nanchangmycin was shown to actively inhibit entry of CHIKV, DENV and WNV into human cells. During the same year of its discovery in 2017, Estoppey et al. identified a fungal lipopeptide, cavinafungin (**33**), from *Colispora* *cavincola* with potent activity of nanomolar range against ZIKV and DENV 1–4 serotypes, albeit with less inhibitory efficacy against CHIKV [85]. The selective antiviral activity of compound **33** was demonstrated to largely stem from its inhibition of endoplasmic reticulum host signal peptidase (ER-SPase) activity that consequently impairs viral polyprotein processing. Another screen of myxobacteria extracts, which was conducted in Germany, led to isolation of a polyketide soraphen A (SorA) (**34**) that targeted host acetyl-CoA carboxylase, a key lipid biosynthetic enzyme. SorA inhibits DENV in vitro at EC_50_ of 4.7 nM and reduces the viral load in vivo with promising pharmacological profile [86]. Prochnow et al., from the same research group, recently isolated labyrinthopeptins A1 (**35**) and A2 (**36**) from actinomycete *Actinomadura* *namibiensis* DCM 6313 [87]. These compounds **35** and **36**, which bind to lipid phosphatidylethanolamine on the viral membranes, exert broad-spectrum antiviral activities against diverse human viruses including DENV, CHIKV, ZIKV and WNV at low micromolar to nanomolar ranges. A high-throughput screening of 774 FDA-approved drugs for anti-ZIKV chemotherapy by repurposing resulted in identification of; the antiparasitic ivermectin (**5**), an inosine-5′-monophosphate (IMPDH) inhibitor mycophenolic acid (MPA, **37**), the cyclophilin inhibitor cyclosporine A (**38**), and a lipopeptide daptomycin (**39**) [88]. Besides the aforementioned activities as anthelmintic, antimalarial, antifilarial and mosquitocidal, it is not surprising for the wonder drug ivermectin (**5**) to inhibit arboviruses, including not only ZIKV (EC_50_ 1–10 µM) but also DENV, WNV and CHIKV (EC_50_ 0.6 µM) by interacting with non-structural (ns) helicase protein 3 [89, 90]. The IMPDH inhibitor, MPA (**37**), was first discovered from *Penicillium* *stoloniferum* during 1893–1896 and approved for reducing transplantation rejection. In 2002, Diamond and colleagues reported the anti-DENV activity of MPA whose mechanism of action is through impairment of viral genome replication; in addition, a similar antiviral activity was later reported for CHIKV and ZIKV (EC_50_ 0.1–1 µM) [88, 91]. Likely blockage to directly access purines from the host cells through IMPDH inhibition slowing viral replication is perhaps the possible mechanism of action of MPA. Like MPA, cyclosporine A (**38**; a fermentation product of *Trichoderma* *polysporum*) inhibits viral RNA synthesis, but through interference of viral NS5 protein interaction with human cyclophilins. Such protein–protein interaction interference perturbs viral replication affording cyclosporine A antiviral activity against DENV and ZIKV [92]. In the case of daptomycin (**39**) from *Streptomyces* *roseosporus*, its antiviral mechanisms against ZIKV are yet unknown but postulated to interfere with viral cell entry. Whilst Barrows et al. in vitro screen for daptomycin’s anti-ZIKV activity was exciting at EC_50_ 0.1–1 µM, this efficacy was unfortunately lost and invalidated in infected *Aedes* mosquitoes [93].

**Fig. 5:**
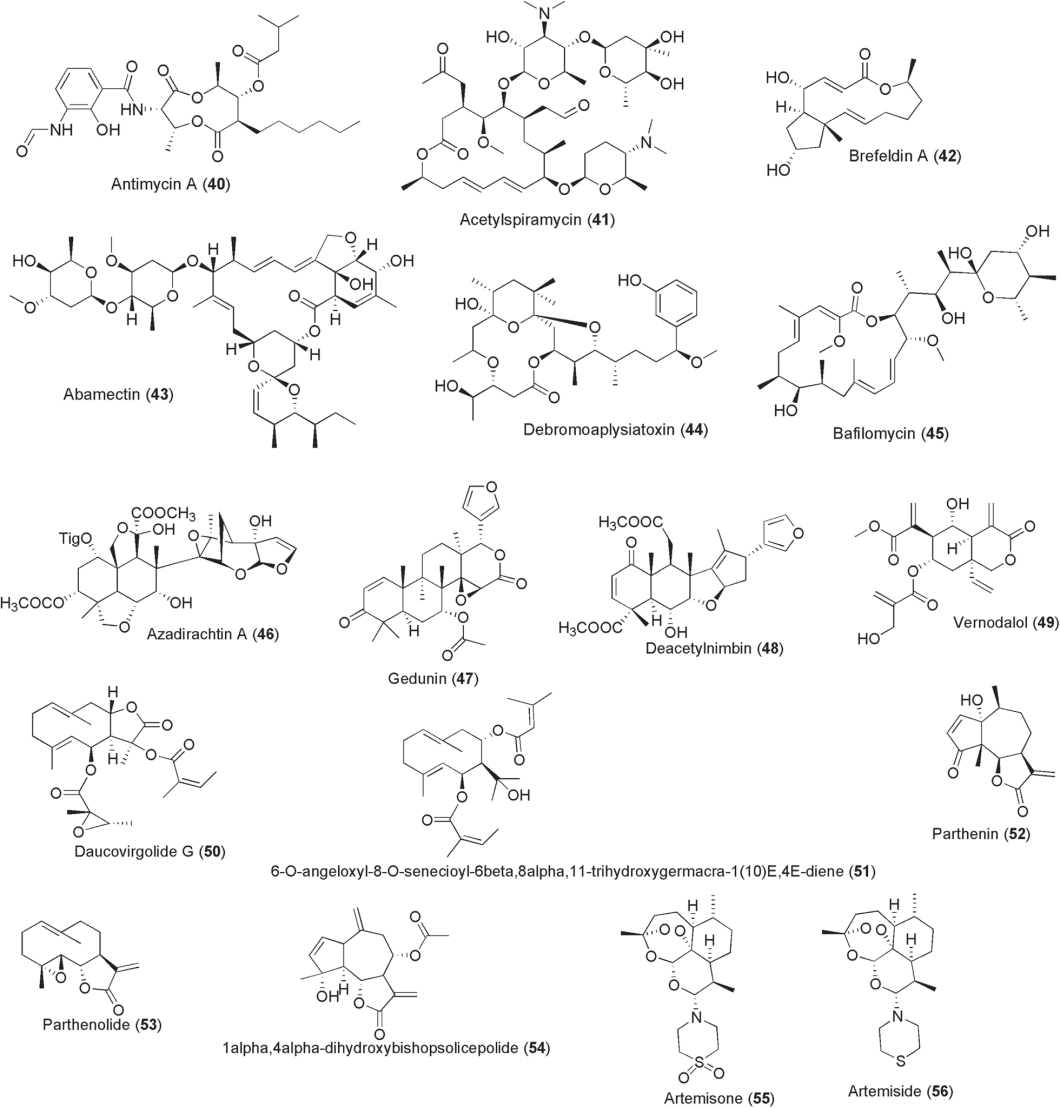
Chemical structures of compounds **40**–**56**. Antimycin A1a (**40**), acetylspiramycin (**41**), brefeldin a (**42**), abamectin (**43**), debromoaplysiatoxin (**44**), bafilomycin (**45**), azadirachtin a (**46**), gedunin (**47**), deacetylnimbin (**48**), vernodalol (**49**), daucovirgolide g (**50**), 6-*o*-angeloxyl-8-*o*-senecioyl-6β,8α,11-trihydroxygermacra-1(10)*e*,4*e*-diene (**51**), parthenin (**52**), parthenolide (**53**), 1α,4α -dihydroxybishopsolicepolide (**54**), artemisone (**55**), artemiside (**56**)

Additional compounds with anti-arboviral activity from microbes are acknowledged. Antimycin A1a [**40**; produced by *Streptomyces* *kaviengensis* (Fig. 5)] is a potential anti-DENV agent identified by high-throughput screening of microbial compounds. Antimycin A exerts antiviral activity at IC_50_ 80 nM [94]. Whilst its known cellular mechanism is through binding the Qi site of cytochrome c reductase and inhibition of oxidative phosphorylation, the precise antiviral mechanism is to date unclear. Further interrogation of the same screen yielded acetylspiramycin (**41**; from *Streptomyces* *ambofaciens*) with anti-DENV activity at IC_50_ 0.91 µM. A polyketone from *Penicillium* sp., brefeldin A (**42**), inhibits DENV at IC_50_ 54.6 nM [95]. Apart from ivermectin, a related macrolide abamectin (**43**) from fermentation of *Streptomyces* *avermitilis* identified by a high-throughput screen performed by Varghese et al. was shown to inhibit CHIKV replication, besides other flaviviruses, at EC_50_ 1.5 µM [90]. Antibiotics, doxycycline (**25**) and azithromycin (**28**), are also potential anti-arbovirals targeting CHIKV and ZIKV infections, respectively [96, 97]. Debromoaplysiatoxin (**44**), and its 3-methoxy derivative have been isolated from Singaporean *Trichodesmium* *erythraeum* (TLTY/PSK/001). These compounds inhibit CHIKV replications in vitro at IC_50_ 1.3 and 2.7 µM [98].

Perhaps the more exciting bioactivity exhibited by these compounds is the blockade of viral passage through insect vectors after ingestion of viraemic blood meals, in addition to their in vitro antiviral activity in mammalian cells. Here we should highlight the works by Dimopoulos’ group. For instance, Kang et al., demonstrated that thoracic microinjection of a macrolide bafilomycin [**45**; an inhibitor of vacuolar H + -ATPase (vATPase) from fermentation products of *Streptomyces* spp.] and mycophenolic acid (**37**; MPA) block DENV-2 infection in *Ae*. *aegypti* [99]. The authors demonstrated that microinjection of either **45** (5 µM) or **37** (250 µM) a day prior to ingestion of DENV-2 viraemic blood meal led to inhibition of viral titres in the salivary glands by 90% and 83%, respectively, at day 14 post-infection. These findings are not different from the recently reported inhibitory efficacy of these compounds against ZIKV in C6/36 cells and mosquito midguts [93].

### Plant-derived compounds

Over the years plants have been indispensable sources of drug-like molecules that are sought as curatives and/or novel scaffolds for drug lead development against various human disease pathogens. Apart from their use in clinical treatment, innovative exploration of these bioactives as promising pathogen transmission blockers has lately gained remarkable traction. Moreover, an emerging translational approach to control disease transmission motivated by exploitation of female insect vectors’ sugar foraging behaviour from randomly selected host plants is explorable for novel interventions [20]. From this ecological perspective, the female disease vectors are evidently reported to feed on particular plant families, harbour host plant tissue DNA as foraging evidence or be manipulated by developing pathogens for increased plant sugar uptake [100, 101]. Laboratory and field studies [102–109] show both native and invasive alien host plant tissues or secretions being ingested by phlebotomine sand flies, arboviral *Aedes* mosquitoes, *Anopheles* *gambiae* and triatomine bug *Rhodnius* *prolixus*. Table 1 summarizes these identified host plants foraged by various insect vectors.

**Table 1:** Examples of the host plants ingested by various disease vectors

Serial number	Host plants^a^	Disease vector	References
1	*Prosopis* *juliflora* (Fabaceae), *Vachellia* *tortilis* (Fabacae), *V.* *nilotica* (Fabacae)*,* *Senegalia* *laeta* (Fabacae), *Cannabis* *sativa* (Cannabaceae)	Phlebotomine sand flies	[104, 105]
2	*Pithecellobium* *dulce* (Fabaceae), *Senna* *uniflora* (Fabaceae), *Hibiscus* *heterophyllus* (Malvaceae), *Opuntia* *ficus*-*indica* (Cactaceae), *Prosopis* *juliflora* (Fabaceae), *Hibiscus* *rosasinensis* (Malvaceae), *Azadirachta* *indica* (Meliacae), *Zea* *mays* (Poaceae), *Vigna* *unguiculata* (Fabaceae), *Malva* *parviflora* (Malvaceae), *Acacia* spp. (Fabaceae)	*Aedes* spp.	[100, 103, 110]
3	*Solanum* *lycopersicum* (Solanaceae)	Triatomine *Rhodnius* *prolixus*	[108]
4	*Prosopis* *juliflora* (Fabaceae), *Parthenium* *hysterophorus* (Asteraceae), *Senna* *occidentalis* (Fabaceae), *Senna* *alata* (Fabaceae), *Senna* *tora*, (Fabaceae), *Ricinus* *communis* (Euphorbiaceae), *Leonotis* *nepetifolia* (Lamiaceae), *Bidens* *pilosa* (Asteraceae), *Senna* *didymobotrya* (Fabacae), *Tecoma* *stans* (Bignoniaceae), *Acacia* *macrostachya* (Fabaceae), *Faidherbia* *albida* (Fabaceae), *Boscia* *angustifolia* (Capparaceae), *Ziziphus* *jujuba* (Rhamnaceae), *Mangifera* *indica* (Anacardiaceae), *Delonix* *regia* (Fabaceae), *Thevetia* *neriifolia*, *Senna* *siamea* (Fabaceae), *Cassia* *sieberiana* (Fabaceae)	*An.* *gambiae*	[100, 109, 111, 112]

^a^Host plants were validated by PCR targeting chloroplast DNA using gene-specific primers: *matK*, *rbcL* and *trnH*-*psbA*, and also through chemical olfactory attractiveness

Besides the pervasive quest for plant sugars, ingestion of other bioactive secondary metabolites is likely to occur and consequently have variable detrimental effects on the development of infectious pathogens harboured in vector’s midguts and salivary glands [105, 113–115]. Although the above list in Table 1 is not exhaustive, because geographical sites and seasons of insect vector collection could influence plant foraging diversity, these studies inform a feasible starting point in the search for chemoprotective compounds. Exemplar agents and others are described below.

#### Terpene derivatives

Natively, neem trees (*Azadirachta* *indica*; Meliaceae) are among the most sought sources of plant-derived remedies at primary care level. Neem has been widely characterized and known for its bioactive terpene derivatives, azadirachtin A (**46**), gedunin (**47**), nimbolide, nimbin, salannin, azadirone, azadiradione, deacetylnimbin (**48**), etc., as well as its standardized alcoholic formulation, NeemAzal^®^ [116, 117]. In 2002, Billker et al. reported remarkable distortions of mitotic microtubule arrays and axonemes in activated male gametes of *P.* *berghei* by compound **46** [118]. This cytoskeleton assembly disruption impaired exflagellations, subsequent fertilization and ookinete development. Subsequent studies by Annete Habluetzel’s team have demonstrated in vitro and in vivo *Plasmodium* transmission-blocking activities by neem terpene compounds [119–121]. In this regard, the standardized NeemAzal^®^ (34% azadirachtin A, 4% salannin, 2% nimbins) reduced the number of zygotes developing into mature ookinetes and exerted a 100% blockade of oocysts in *An*. *stephensi* at 50 mg/kg in vivo dose [121]. Synergistic action of NeemAzal^®^ constituents afforded a stronger activity against early sporogonic stages of *Plasmodium* compared to azadirachtin A alone [120]. From the seed kernels, Tapanelli et al. isolated various limonoids. A thermally and chemically stable deacetylnimbin (**48**, an analogue of nimbin) was highlighted as a potential inhibitor of early sporogonic stages achieving a 100% parasite clearance at 100 µM [119]. Gedunin (**47**) is a potent plasmodial Hsp90 inhibitor and has been identified among the promising inhibitors of *Plasmodium* liver stages with prospective prophylactic efficacy [69]. Other compounds with potential malaria transmission-blocking activity have also emerged from Habluetzel’s research team. Abay et al. [122] identified a sesquiterpene lactone, vernodalol (**49**), from *Vernonia* *amygdalina* (Asteraceae) leaves modestly acting against *P.* *berghei* zygotes and ookinetes at IC_50_ 18.7 µM, but did not impair microgamete formation even when tested at high concentration of 50 µM. Germacranolide sesquiterpenoids, daucovirgolides A–L and polyoxygenated germacranes have been isolated from Tunisian plants of *Daucus* genera (Apiaceae), *D.* *virgatus* and *D.* *carota* [123–125]. Remarkable *P.* *berghei* ookinete formation inhibitory activities were noted for daucovirgolide G (**50**) (92% at 50 µg/ml; IC_50_ 17.5 µM) and 6-*O*-angeloxyl-8-*O*-senecioyl-6β,8α,11-trihydroxygermacra-1(10)*E*,4*E*-diene (**51**) (86.4% at 50 mM; IC_50_ 96.4 µM), without a general cytotoxicity. Whilst no apparent defined mechanism or biological target has been identified yet, the observed activity is hypothesized to result from the intact endocyclic double-bond system of these compounds [124].

**Fig. 6:**
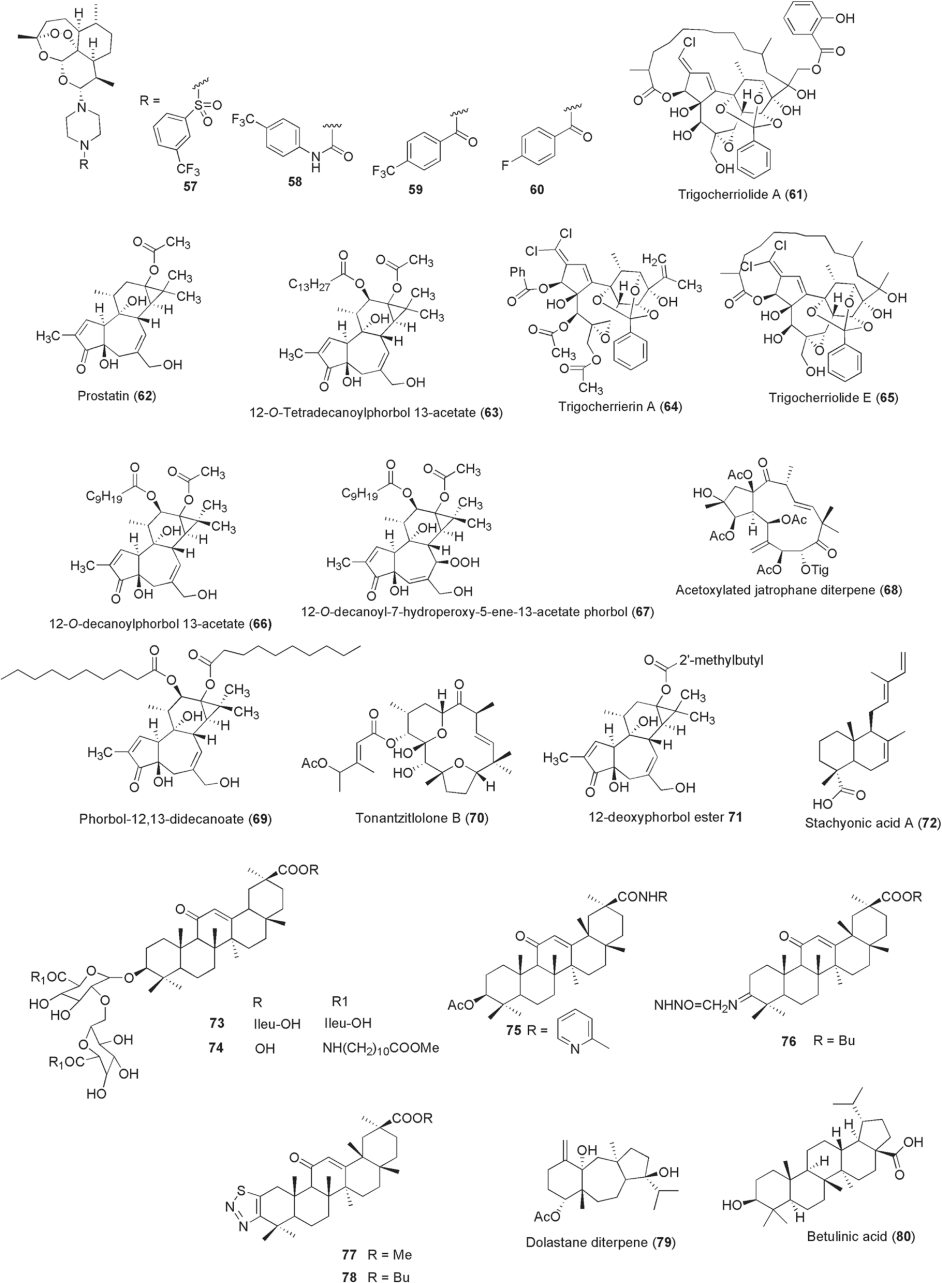
Chemical structures of compounds **57**–**80**. 10-Aminoartemisinin compound (**57**), 10-aminoartemisinin compound (**58**), 10-aminoartemisinin compound (**59**), 10-aminoartemisinin compound (**60**), trigocherriolide A (**61**), prostratin (**62**), 12-*O*-tetradecanoylphorbol 13-acetate (**63**), trigocherrierin A (**64**), trigocherriolide E (**65**), 12-*O*-decanoylphorbol 13-acetate (**66**), 12-*O*-decanoyl-7-hydroperoxy-phorbol-5-ene-13-acetate (**67**), (*2R,3R,4S,5R,7S,8R,13R,15R*)-3,5,7,15-tetraacetoxy-2-hydroxy-8-tigloyloxy-9,14-dioxojatropha-6(17),11*E*-diene (**68**), phorbol-12,13-didecanoate (**69**), tonantzitlolone B (**70**), 12-deoxyphorbol-13(2"-methyl)butyrate (**71**), stachyonic acid a (**72**), compound **73**, compound **74**, compound **75**, compound **76**, compound **77**, compound **78**, ((*4r,9s,14s*)-4α-acetoxy-9β,14α-dihidroxydolast-1(15),7-diene (**79**), betulinic acid (**80**)

Parthenin (**52**), a major sesquiterpene lactone from a mosquito preferred host plant, the invasive *Parthenium* *hysterophorus* (Asteraceae), is well tolerated by female mosquito vectors without any apparent tissue toxicity [109]. Motivated in part by this initial finding, Balaich and colleagues examined parthenin’s inhibitory effects against transmissible sporogony stages of *P.* *falciparum* alongside a structurally related parthenolide (**53**) from *Tanacetum* *parthenium* (Asteraceae). The authors noted decreased *Plasmodium* oocyst densities of 40–80% on offering mosquitoes 6.25 µg/ml parthenin in gametocytemic blood meal and a complete clearance at doses between 50–100 µg/ml. Similar activity was exerted by parthenolide at 40 nM to 4 µM and poised to occur through inactivation of stage V gametocytes, inhibition of microgamete exflagellation and impaired ookinete maturation [126]. Another gametocytocidal guaianolide sesquiterpenoid, 1α,4α -dihydroxybishopsolicepolide (**54**), was recently isolated from a South African plant of Asteraceae family *Artemisia* *afra* (Asteraceae). Compared to its activity against early gametocytes, compound **54** was demonstrated to exert better cidal activity against the late-stage IV/V gametocytes (IC_50_ 6.3 µM) [127]. This is however in contrast to derivatives of artemisinin from *Artemisia* *annua* (Asteraceae): dihydroartemisinin (DHA), artemether and artesunate with relatively poor activity against late-stage IV/V gametocytes [128]. Besides their rapid clearance of asexual stages, they also potently kill early stage I-III gametocytes reducing gametocyte carriage. But, failure to clinically clear stage IV/V gametocytes by these artemisinin derivatives promotes the transmission of *Plasmodium* to mosquitoes, including parasites from artemisinin-based combination therapy (ACT) resistance backgrounds [129, 130]. Inspired to reverse this challenge into better antimalarials, Coertzen et al. [131] and Wong et al. [132] have developed artemisone (**55**), artemiside (**56**) and 10-aminoartemisinins **57**–**60** designed from artemisinin skeleton (Fig. 6). These compounds **55**–**60** exhibit preferential nanomolar activity against late-stage IV/V gametocytes (IC_50_ 0.04–42.4 nM), without being overshadowed by artemisinin’s PfKelch-13 C580Y mutation genotypes.

Antiviral activities of terpene derivatives against various arboviruses are reported, particularly in experiments utilizing in vitro conditions. An oxygenated diterpenoid, trigocherriolide A **(61**), was isolated alongside other compounds from the bark of a New Caledonian plant *Trigonostemon* *cherrieri* (Euphorbiaceae) in 2012 [133]. A relatively strong inhibitory activity of compound **61** (IC_50_ 3.1 µM) for DENV NS5 RdRp was reported. In the same year, two plant-derived phorbol esters, prostratin [**62**; from *Homalanthus* *nutans* (Euphorbiaceae)] and 12-*O*-tetradecanoylphorbol 13-acetate (**63**), were reported to selectively inhibit CHIKV replication at EC_50_ values of 2.6 µM and 2.9 nM, respectively [134]. Bourjot et al. later isolated unusually chlorinated daphane diterpenoid orthoesters (DDO) from the leaves of *Trigonostemon* *cherrieri*, among them trigocherrierin A (**64**) and trigocherriolide E **(65**). Using a viral cell-based assay, the authors reported potent inhibition of CHIKV by compound **64** (EC_50_ 0.6 µM), with similar bioactivity exhibited by **65** (EC_50_ 0.7 µM) [135]. From the leaves of another Euphorbiaceae plant, *Croton* *mauritianus*, Corlay et al. isolated alongside other compounds two promising anti-CHIKV tigliane diterpenes, 12-*O*-decanoylphorbol 13-acetate (**66**) and 12-*O*-decanoyl-7-hydroperoxy-phorbol-5-ene-13-acetate (**67**). Compounds **66** and **67** inhibited CHIKV-induced cell death at EC_50_s of 2.4 µM and 4 µM, respectively [136]. In the same spirit of finding anti-CHIKV inhibitors, Nothias-Scaglia et al. identified a potent acetoxylated jatrophane diterpene (*2R,3R,4S,5R,7S,8R,13R,15R*)-3,5,7,15-tetraacetoxy-2-hydroxy-8-tigloyloxy-9,14-dioxojatropha-6(17),11*E*-diene (**68**) from a Mediterranean *Euphorbia* *amygdaloides* (EC_50_ 0.76 µM) [137]. In a follow-up study from the same group in 2015, 29 commercially available natural diterpenoids were screened against CHIKV and HIV replications. This effort led to identification of a potent inhibitor agent phorbol-12,13-didecanoate (**69**) with anti-CHIKV activity (EC_50_ 6 nM) [138].

In addition, tonantzitlolone-type diterpenes were isolated from stem barks of Euphorbiaceae plant *Stillingia* *lineata* collected in Reunion Island and screened against CHIKV. Among the compounds, Techer et al. [139] reported 4′-acetoxytonantzitlolone (**70**; tonantzitlolone B) endowed with a promising anti-CHIKV activity (EC_50_ 7 µM). From the leaves of the same plant, a more potent tigliane diterpenoid 12-deoxyphorbol-13(2"-methyl)butyrate (**71**) was isolated (anti-CHIKV, EC_50_ 1.2 µM) [140]. A labdane diterpene stachyonic acid A (**72**) was isolated in 2019 from *Basilicum* *polystachyon* (Lamiaceae) [141]. By using a DENV plaque-reduction neutralization (PRNT) assay, compound **72** exerted an antiviral activity of IC_50_ 1.4 µM relative to less potent andrographolide (IC_50_ 51 µM). Elsewhere, antiviral triterpenoid compounds have been reported from the roots of licorice herb *Glycyrrhiza* *glabra* (Fabaceae). Unlike the parent compound glycyrrhizic acid that exerts anti-DENV-2 activity (IC_50_ 8.1 µM), its derivatization through chemical conjugation with isoleucine and 11-aminoundecanoic acid methyl ester resulted in potent compounds **73** (IC_50_ 1.3 µM) and **74** (IC_50_ 1.2 µM) [142]. Through a similar strategy, a more recent study reported derivatives of a pentacyclic triterpenoid, glycyrrhetinic acid from *G.* *gabra*, with potent anti-ZIKV replication activity [143]. The resultant compounds **75**–**78** had IC_50_ values of 0.13 µM, 0.55 µM, 0.29 µM and 0.56 µM, respectively. From a marine brown seaweed (*Canistrocarpus* *cervicornis*) collected from Praia do Velho in Angra dos Reis (Brazil), a dolastane diterpene ((4R,9S,14S)-4α-acetoxy-9β,14α-dihidroxydolast-1(15),7-diene; **79**) was isolated and reported to inhibit ZIKV (EC_50_ 0.95 µM) and CHIKV (EC_50_ 1.3 µM) [144]. Elsewhere the triterpenoid betulinic acid (**80**) displayed an anti-DENV-2 activity at IC_50_ 0.95 µM, with a specific inhibition exerted at viral RNA replication step of other DENV serotypes (DENV-1,3,4; IC_50_ 0.9–1.84 µM) and ZIKV (IC_50_ 2.45 µM) [145].

**Fig. 7:**
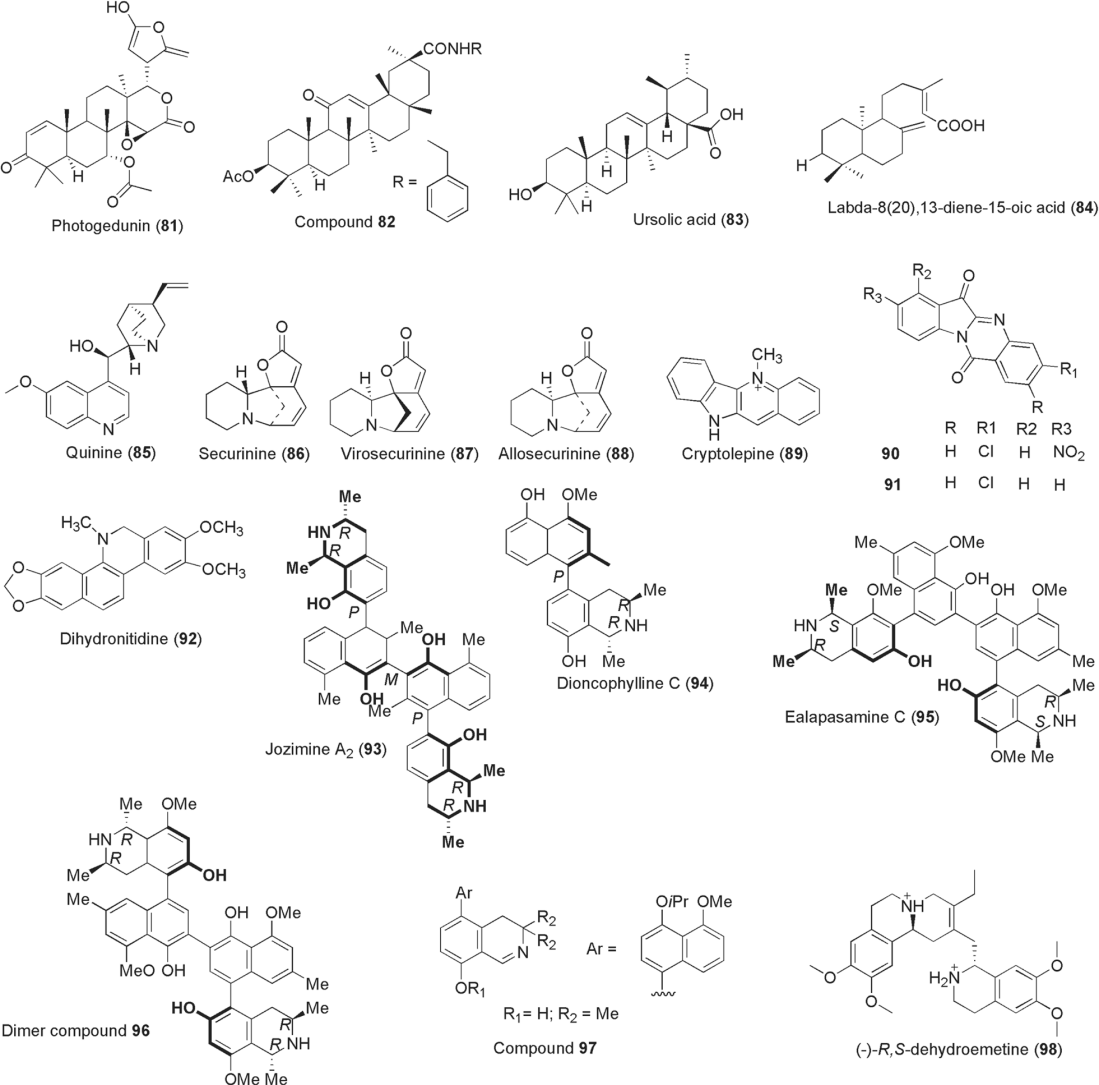
Chemical structures of compounds **81**–**98**. Photogedunin (**81**), analog compound **82**, ursolic acid (**83**), labda-8(20),13-diene-15-oic acid (**84**), quinine (**85**), securinine (**86**), virosecurinine (**87**), allosecurinine (**88**), cryptolepine (**89**), 3-chloro-8-nitro-tryptanthrin, 3-chloro-8-nitro-indolo [2,1-*b*] quinazoline-6,12-dione (nt1) (**90**), 3-chloro-indolo [2,1-*b*] quinazoline-6,12-dione (t8) (**91**), dihydronitidine (**92**), jozimine a_2_ (**93**), dioncophylline c (**94**), ealapasamine c (**95**), dimer compound **96**, compound **97**, (−)-*R,S*-dehydroemetine (**98**)

The compounds gedunin (**47**) and photogedunin (**81**; Fig. 7) were isolated from ethyl acetate fractions derived from the fruits of naturally growing *Xylocarpus* *granatum* (Meliaceae). Evaluation of these compounds against filarial worms, *B.* *malayi*, resulted in complete immobilization and macrofilaricidal activity at IC_50_ values of 0.239 µg/ml and 0.213 µg/ml, respectively [146]. Kalani et al. isolated and derivatized glycyrrhetinic acid that exhibited potential antifilarial activity against the microfilariae (IC_50_ 1.2 µM) but was inactive against the adult worms of *B.* *malayi*. The authors reported an improved amide analogue **82** with lesser potency against microfilariae (IC_50_ 2.2 µM) but active against adult worms at IC_50_ of 8.8 µM [147]. At a concentration of 10 µg/ml ursolic acid [**83**; isolated from ethyl acetate fraction of *Nyctanthes* *arbortristis* (Oleaceae)], about 84.15% reduction of *W.* *bancrofti* microfilariae viability was achieved through redox imbalance [148]. In 2016, antifilarial activity of compounds isolated from *Taxodium* *distichum* (Cupressaceae) collected from Palampur, India, was investigated [149]. Among the compounds identified, the diterpenoid labda-8(20),13-diene-15-oic acid (**84**) exerted a 100% reduction in motility of *B.* *malayi* microfilariae and adult worms, killed > 80% adult worms in an infected mouse model (dose: 100 mg/kg for 5 days) and sterilized > 36% female worms.

#### Alkaloids

Various plant-derived compounds of the alkaloid class show profound activities against infectious pathogens in the context of blocking disease transmissions between hosts. In view of this, the first ever discovered antimalarial compound quinine (**85**) has shown cidal effects on the early gametocytes, but weak activity against mature gametocytes of *Plasmodium* *falciparum* from various screening platforms [128, 150]. Yet, quinine could effectively kill mature gametocytes of *P.* *vivax* and *P.* *malariae*, as well as reduce *P.* *falciparum* oocysts numbers when provided at higher concentrations of EC_50_ 642 ng/ml [151, 152]. Attempts to find other potential *Plasmodium* transmission-blocking agents utilizing a fragment-based screening approach of natural products afforded the identification of three compounds based on securinine [153]. (−)-Securinine is an alkaloid sourced from two Phyllanthaceae plants *Securinega* *suffruticosa* and *Phyllanthus* *niruri*. Securinine-related compounds **86**–**88** from the fragment screen inhibited > 80% *Plasmodium* gametocyte viability at 100 µM through an allosteric binding of 2′-deoxyuridine 5′-triphosphate nucleotidohydrolase (PfdUTPase). From the West African antimalarial herbal plant *Cryptolepis* *sanguinolenta* (Periplocaceae), its main alkaloid constituent cryptolepine (**89**) was demonstrated to exert late-stage NF54 gametocytocidal activity at IC_50_ 1.97 µM [154].

Onambele et al. [155] designed and synthesized various derivatives of a (indolo-2,1-*b*)-quinazoline-6,12-dione [tryptanthrin; derived from *Isatis* *tinctoria* (Brassicaceae)]. Among the synthesized compounds, two derivatives designated as NT1 (**90**; 3-chloro-8-nitro-tryptanthrin, 3-chloro-8-nitro-indolo [2,1-*b*] quinazoline-6,12-dione) and T8 (**91**; 3-chloro-indolo [2,1-*b*] quinazoline-6,12-dione) emerged to confer 100% inhibition of gametocyte maturation when tested at their IC_90_ concentrations. Despite this promising gametocytocidal activity, the compounds unfortunately had weak inhibition of microgamete exflagellations with only 20% for NT1 [155]. Goodman et al. isolated dihydronitidine **(92**) alongside other compounds from the stem bark of *Zanthoxylum* *heitzii* (Rutaceae). When tested for in vitro *P.* *berghei* ANKA ookinete conversion inhibitions, dihydronitidine exerted a more potent activity at IC_50_ 0.59 µg/ml compared to heitziquinone at IC_50_ 6.2 µg/ml [156]. Moreover, following the successful isolation of various potent anti-infective naphthylisoquinoline alkaloids from rare lianas of Ancistrocladaceae and Dioncophyllaceae by a team led by Gerhard Bringmann, Moyo et al. subsequently tested for their gametocytocidal activity profiles. As a result, Jozimine A_2_ (**93**), dioncophylline C (**94**), ealapasamine C (**95**), dimer compound (**96**) and compound **97** tested at 2 µM inhibited male gamete exflagellations between 73 and 100% [157]. With exception of compounds **94** and **97** (not tested), potent gametocytocidal activity was reported against early and late gametocytes: jozimine A_2_: early gametocytes IC_50_ 0.375, late gametocytes IC_50_ 0.511 µM; ealapasamine C: early gametocytes IC_50_ 0.545, late gametocytes IC_50_ 0.889 µM; dimer compound **96**: early gametocytes IC_50_ 0.542, late gametocytes IC_50_ 0.623 µM [157]. In the same period in 2020, Panwar et al. reported a synthetic analogue of emetine dihydrochloride, (-)-*R,S*-dehydroemetine (**98**), identified through a drug repositioning strategy and lead optimization. Emetine dihydrochloride hydrate is derived from *Psychotria* *ipecacuanha* (Rubiaceae). The emetine derivative compound **98** was demonstrated to possess potent inhibition against asexual parasite stages and prevented activated *P.* *falciparum* NF54 gametocytes from progressing into gametes in a dual gamete formation assay at IC_50_ 0.43 µM (male gametocytes) and 1.04 µM (female gametocytes) [158].

**Fig. 8:**
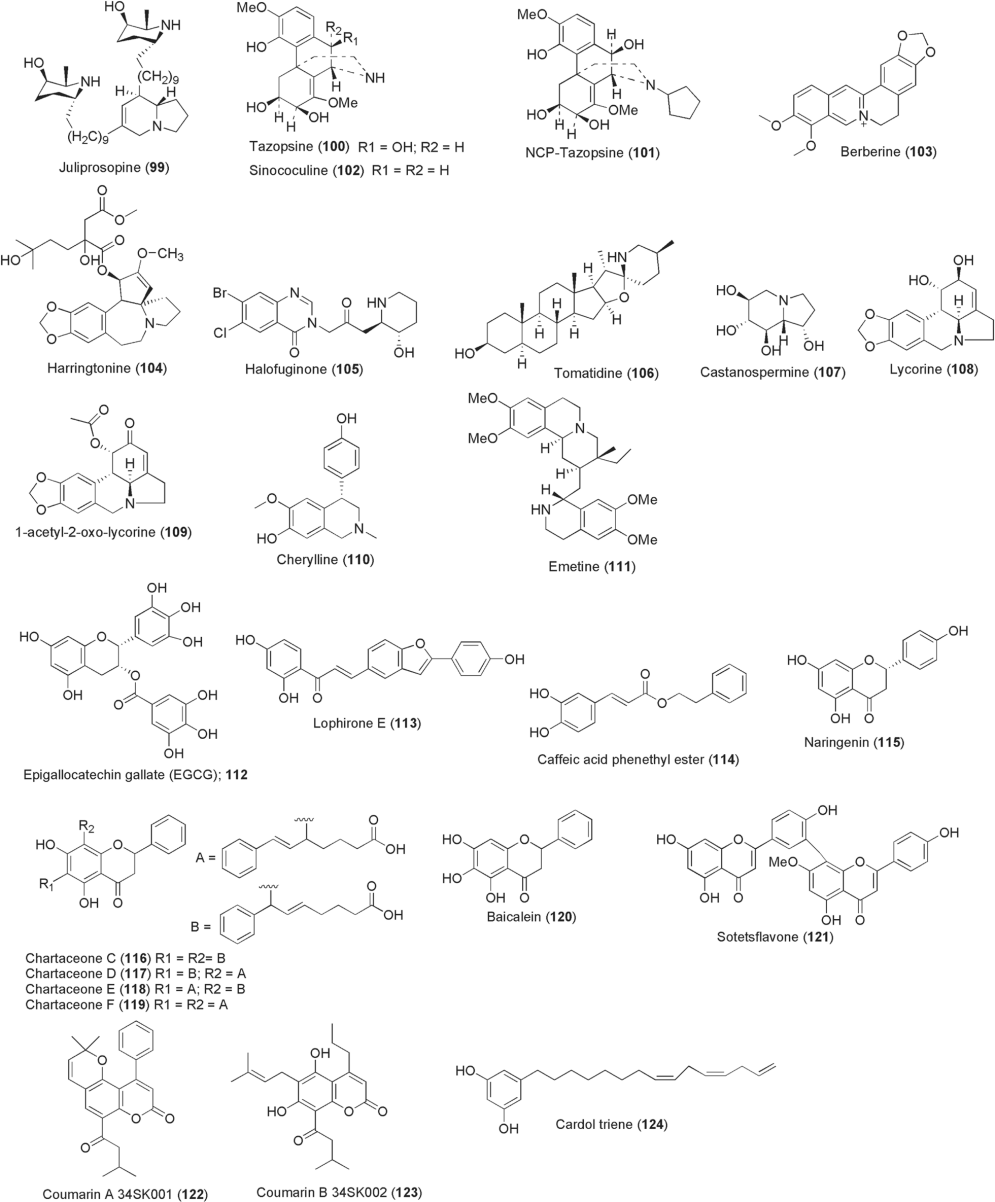
Chemical structures of compounds **99**–**124**. Juliprosopine (**99**), tazopsine (**100**), NCP-tazopsine (**101**), sinococuline (**102**), berberine (**103**), harringtonine (**104**), halofuginone (**105**), tomatidine (**106**), castanospermine (**107**), lycorine (**108**), 1-acetyllycorine analogue (**109**), cherylline (**110**), emetine (**111**), epigallocatechin gallate (**112**), lophirone e (**113**), caffeic acid phenethyl ester (**114**), naringenin (**115**), chartaceone c (**116**), chartaceone d (**117**), chartaceone e (**118**), chartaceone f (**119**), baicalein (**120**), sotetsflavone (**121**), coumarin a 34sk001 (**122**), coumarin b 34sk002 (**123**), cardol triene (**124**)

Mosquitoes have been reported to commonly feed on the invasive plant *Prosopis* *juliflora* (Fabaceae) (Table 1) for sugar acquisition. The *Prosopis* plant is a reliable source of indolizidine alkaloids, majorly the juliprosopine (**99**; Fig. 8). We recently reported findings from our study, which demonstrated potent gametocytocidal activity of juliprosopine against late-stage IV/V gametocytes of *Plasmodium* clinical isolates (IC_50_ < 1 µM) (patent no. KE/P/2020/3643) [159]. Compound **99** further strongly impaired sexual conversions, with no observable young NF54 gametocytes on day 5–7 post-induction, and killed developing ookinetes in vitro without lethal effects on survival of female mosquitoes (patent no. KE/P/2020/3643). Elsewhere, Carraz et al. reported a morphinan alkaloid from stem bark of a Madagascan Menispermaceae plant *Strychnopsis* *thouarsii*. Evaluations performed against *Plasmodium* *yoelii* and *P.* *falciparum* liver stages led to the identification of tazopsine (**100**), with a promising cidal activity (*P.* *yoelii*; IC_50_ 3.1 µM, IC_90_ 6.3 µM; *P.* *falciparum* IC_50_ 4.2 µM, IC_90_ 18.3 µM). Following an establishment of its toxicity in mice and cultured cells, modification through N-alkylation of tazopsine resulted in NCP-tazopsine (**101**) with improved therapeutic index, low cellular toxicity and IC_50_ values, *P.* *yoelii* (3.3 µM) and *P.* *falciparum* (42.4 µM) [160]. Follow-up study on *S.* *thouarsii* yielded, among other morphinan compounds, sinococuline (**102**), displaying slightly less but comparable activity against *P.* *yoelii* liver stages to tazopsine (IC_50_ 4.5 µM) [161].

In vitro studies highlight plant-derived alkaloids as excellent antiviral scaffolds despite not being tested in arboviral mosquito vectors. The antimalarial quinine (**85**) is a ten-fold more potent inhibitor of CHIKV replication (IC_50_ 0.1 µg/ml) compared to its derivative chloroquine (CQ) (IC_50_ 1.1 µg/ml) [162]. A relatively nontoxic isoquinoline berberine **(103**) identified through a high-throughput screen targets virus-induced mitogen-activated protein kinase (MAPK) signalling to inhibit CHIKV replication (EC_50_ 1.8 µM) [90, 163]. Furthermore, a recent study [164] reported the ability of berberine to impair CHIKV nucleocapsid assembly at later stages of the viral life cycle, with decreased infectivity of viral particles produced from the treated cells suggesting dual mechanisms of its antiviral activity. Kaur et al. screened a 502 natural product compound library and identified the highly potent anti-CHIKV agent harringtonine [**104**; derived from *Cephalotaxus* *harringtonia* (Taxaceae)] (EC_50_ 0.24 µM) [165]. Harringtonine inhibited CHIKV after cell entry 6 h post-infection by targeting viral protein synthesis. Through targeting host translational machinery hijacked by invading viruses, Hwang et al. demonstrated potent inhibition of DENV and CHIKV by a synthetic derivative of plant-derived febrifugine (halofuginone; **105**) at 100 nM [166]. A recently identified steroidal alkaloid tomatidine (**106**; isolated from leaves and stem of unripe tomatoes) reduced CHIKV particle production (93.7%) in various mammalian cells at additional 2 h post-infection [167]. Tomatidine achieved its anti-CHIKV activity at EC_50s_ range 1.3–3.9 µM. Also tomatidine inhibits DENV 1–4 in vitro at micromolar EC_50_ range of between 0.82 and 4.87 µM, but is more active against DENV-2 independent of ATF4 transcription factor activation [168].

Among the earliest inhibitors of DENV, the indolizidine alkaloid castanospermine (**107**) isolated from seeds of *Castanospermum* *australe* (Fabaceae) is known to be a potent inhibitor of all DENV 1–4 serotypes [169]. Its antiviral activity stems from inhibiting host cell α-glucosidase activity reducing secretion and infectivity of viral particles. However, when later administered into female *Aedes* mosquitoes via microinjection, castanospermine failed to suppress DENV-2 infectivity [99]. Another potent inhibitor of DENV, as well as YFV, ZIKV, RVFV and WNV, has been derived from the Amaryllidaceae family, lycorine (**108**) and its 1-acetyllycorine analog (**109**). Compound **108** reduces flaviviral titres by up to 10^4^-fold at 1.2 µM and IC_50_ 0.24 µM, while its derivative **109** exerts EC_50_ 0.4 µM [170]. In a recent study by Chen et al. [171], compound **108** compromised ZIKV replication in vitro and in vivo by inhibiting viral RNA replication and protein synthesis at EC_50_ 0.22–0.39 µM. Whilst mechanistic antiviral actions of these compounds **108** and **109** are still unclear, targeted NS4B is possibly the direct interaction protein [172] but also binding of ZIKV RdRp has been postulated [171]. From another Amaryllidaceae plant, namely *Crinum* *jagus* collected from Senegal, antiviral alkaloid cherylline (**110**) was isolated. Cherylline efficiently inhibited DENV and ZIKV at EC_50_ values of 8.8 µM and 20.3 µM by interfering with viral RNA synthesis post-entry step [173]. Elsewhere in a drug repurposing study, the antiprotozoal emetine (**111**) emerged to potently inhibit ZIKV African prototype (ZIKV MR766) infection with an IC_50_ 52.9 nM and completely suppressed ZIKV replication at IC_50_ 8.74 nM. Emetine was identified to exert its antiviral activity by inhibiting ZIKV NS5 polymerase activity and disrupting lysosomal function [174].

Only berberine (**103**) has been reported to be active against lymphatic filarial worms. Li et al. [175] demonstrated that berberine targets *Wolbachial* FtsZ, a cell division protein, inhibiting its GTPase activity. When treated with 10 – 40 µM berberine for 2 days, adult female *B.* *malayi* worms were immobilized and subsequently the microfilariae production was completely stopped.

#### Flavonoids and phenolic derivatives

Flavonoids and phenolics from various plants have been exploited as potential pathogen-blocking agents, with most interrupting arboviral replication cycles. However, very few molecules of this chemical class are presently known to inhibit transmissible stages of *Plasmodium* and lymphatic filarial worms. With reference to malaria, the abundant green tea polyphenol EGCG (epigallocatechin gallate; **112**) was demonstrated in 2010 to kill infective *Plasmodium* sporozoites achieving IC_50_ values of 1.1 µM (6 h) and 0.12 µM (12 h). Mechanistically, EGCG impaired sporozoite gliding motility (IC_50_ 0.14 µM), affecting their infectivity to liver cells through unknown intracellular targets. The sporozoite kill effect of EGCG was reported to be more pronounced through a synergistic addition of membrane permeant digitoxin that decreased overall IC_50_ values to 0.044 µM (6 h) and 0.035 µM (12 h) [176]. From the stem bark of *Lophira* *lanceolata* (Ochnaceae) collected from Burkina Faso, the bioflavonoid lophirone E (**113**) was isolated alongside other compounds from the ethyl acetate fraction phase. Whilst the compound **113** exerted moderate activity against asexual stages of *Plasmodium*, a selectively potent activity against 3D7elo1CBG99 stage V gametocytes at IC_50_ 0.14 µM was reported [177].

Regarding anti-lymphatic filariasis, flavonoids have been investigated for their capacity to abrogate macrofilarial viability and microfilarial productions. Al-Abd et al. reported antifilarial activity of caffeic acid phenethyl ester (**114**) isolated from *Melaleuca* *cajuputi* (Myrtaceae) flowers against *B.* *pahangi* adult worms. In their evaluations, these authors indicated that compound **114** kills adult worms and microfilariae at IC_50_ values of 3.9 µg/ml and 7.5 µg/ml, respectively, while administration of 50 mg/kg compound **114** for 14 days to an infected mouse model reduced circulating microfilariae by 60% and 58% for adult worms. Depletion of *Wolbachia*, demonstrated by reduced *WolbachiaftsZ* gene copy number on treatment, could underlie the observed antifilarial activity [178]. From a screen of six flavonoids against *B.* *malayi* naringenin (**115**) appeared as the most potent filaricidal, immobilizing female adult worms at IC_50_ 2.5 µg/ml and killing 73% of transplanted worms in vivo at 50 mg/kg dose. The molecule was however less effective against microfilariae (IC_50_ 297.3 µg/ml) [179].

**Fig. 9:**
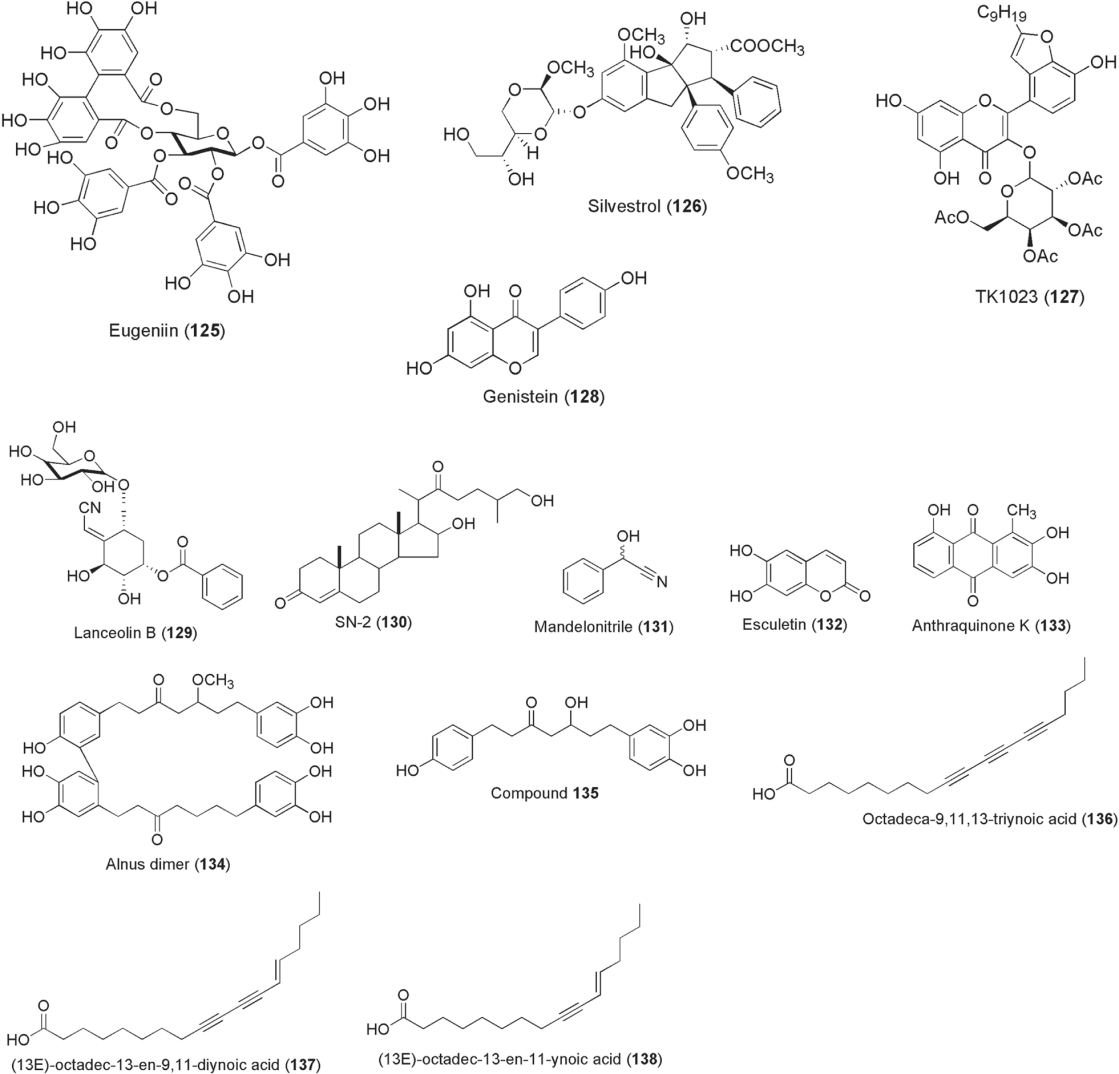
Chemical structures of compounds **125**–**138**. Eugeniin (**125**), silvestrol (**126**), houttuynoid B derivative (tk1023) (**127**), genistein (**128**), lanceolin b (**129**), sn-2 (**130**), mandelonitrile (**131**), esculetin (**132**), anthraquinone k (**133**), alnus dimer (**134**), (*5* *s*)-5- hydroxy-1-(4-hydroxyphenyl)-7-(3,4-dihydroxyphenyl)-3-heptanone (**135**), octadeca-9,11,13-triynoic acid (**136**), (*13e*)-octadec-13-en-9,11-diynoic acid (**137**), (*13E*)-octadec-13-en-11-ynoic acid (**138**)

One of the most widely investigated bioactivites of flavonoids and phenolics in this context is that of anti-arbovirals. However, for the purpose of this review, we focussed on the most potent reported compounds with IC_50_/EC_50_ of < 10 µg/ml and 10 µM. In 2011, Allard et al. isolated various dialkylated flavanones (chartaceones A–F) from the stem bark of *Cryptocarya* *chartacea* (Lauraceae) collected from New Caledonia. Screening these compounds against DENV-2 NS5 RNA-dependent RNA polymerase (RdRp) activity identified chartaceones C–F (**116–119**) as the most potent inhibitors with IC_50_ 1.8–4.2 µM [180]. The bioflavonoid baicalein [**120**; derived from roots of *Scutellaria* *baicalensis* (Labiatae)] exerts potent anti-DENV-2 activity at IC_50_ 1.55 µg/ml [181]. Besides, compound **120** inhibited DENV-3 replication in a virus foci reduction assay at 100 µg/ml (99.78%) and IC_50_ 12.7 µg/ml. The study demonstrated that compound **120** required a short time of contact (0 min) to exert its virucidal activity (62.45%) by blocking viral attachment and cell entry, interfering with infectivity of all DENV 1–4 serotypes [182]. Elsewhere, compounds inhibiting DENV NS5 RdRp activity were isolated from leaves of another New Caledonian plant, *Dacrydium* *araucarioides* (Podocarpaceae), and structure–activity relationships analyzed alongside other bioflavonoids previously obtained from a related plant *D.* *balansae* (Podocarpaceae). From this analysis, the authors pointed out that the number and position of methyl groups on the bioflavonoid moiety as well as the degree of oxygenation of flavonoid monomers influence the anti-DENV bioactivity. The 7"-*O*-methylamentoflavone, sotetsflavone (**121**), from *D.* *araucarioides* with an IC_50_ of 0.16 µM emerged as the strongest DENV-NS5 RdRp inhibitor [183]. Coumarins A 34SK001 (**122**) and B 34SK002 (**123**) isolated from seeds of *Mammea* *americana* (Clusiaceae) collected in the Colombian Caribbean Region (Colombia) were reported to potently inhibit both DENV-2/NG and CHKV-ACol at EC_50_ values: 9.6 and 10.7, 2.6 and 0.5 µg/ml, respectively [184]. Compounds **122** and **123** acted strongly by inhibiting replication of viral genome. Recent studies have further highlighted other potential DENV inhibitors. In 2018, a phenolic lipid cardol triene (**124**) was identified from a structure–activity relationship study of cashew nutshell phenolics as a potential anti-DENV inhibitor. Compound **124** inhibited DENV-2 (EC_50_ 7.13 µM), but also displayed pan-dengue inhibition at EC_50_ values of 5.35–8.89 µM by targeting envelope protein kl loops preventing fusion and infectivity [185]. The compounds 5,7-dihydroxy-2-methylchromone-8C-β-d-glucopyranoside (isobiflorin), 5,7-dihydroxy-2-methylchromone-6C-β-d-glucopyranoside (biflorin) and eugeniin (**125**; Fig. 9) have been isolated from flower buds of cloves [*Syzygium* *aromaticum* (Myrtaceae)]. Only the ellagitannin compound **125** potently inhibited recombinant DENV-2 and -3 NS2BNS3pro complex (IC_50_ 94.7 nM and 7.43 µM, respectively) through a competitive inhibitory mechanism [186].

The polyphenol EGCG (**112**) is a potent viral cell entry inhibitor of CHIKV (IC_50_ 6.54 µg/ml) [187] and inhibits viral infection of CHIKV S27 in U2OS cells at IC_50_ 1.99 µg/ml [188]. Synergistic anti-CHIKV action with suramin against various strains was demonstrated. However, EGCG exerts relatively weak anti-DENV and anti-ZIKV activities. By inhibiting a host cell DEAD-box helicase eIF4A, silvestrol (**126**; derived from *Aglaia* *foveolata*) abrogates CHIKV replication through delayed synthesis of nonstructural and structural proteins in 293 T and NIH3T3 cells at IC_50_ 1.89 nM and 5.06 nM, respectively [189]. Another potent ZIKV cell entry inhibitor was derived from a flavonoid glycoside isolated from *Houttuynia* *cordata* (Sauraceae). A tetra-*O*-acetylated houttuynoid B derivative (TK1023; **127**) strongly reduced ZIKV intracellular and extracellular viral genomes within 48 h post-treatment, achieving EC_50_ values of 1.68 and 1.55 µM against Polynesia and Ugandan strains, respectively [190].

Pre-treatment, and not post-entry treatment, of *Aedes* C6/36 cells with genistein (**128**) at 60 µM impaired WNV replication. Only ~ 25% cells were detected positive for the viral antigen. The anti-WNV activity by compound **128** was through disruption of focal adhesion kinase (FAK) functionality [191].

#### Quinones, steroids, cardiac glycosides and other chemical classes from plants

Motivated by the selective gametocytocidal potency of compound **117** from *Lophira* *lanceolata*, Sore et al. further isolated alongside other compounds two lanceolins of cyanoglucosides class, lanceolin A and B (**129**), containing a cyanomethylene group. Lanceolin B exerted considerable inhibitory activity against *P.* *berghei* early sporogonic stages and ookinete development at IC_50_ values of 12.75 and 10.95 µM, respectively [192]. Besides, steroidal compounds isolated from *Solanum* *nudum* (Solanaceae), SN-1, SN-2 and SN-4, were evaluated for their sporontocidal effects on *P.* *vivax* isolates in *An.* *albimanus.* Administered at doses between 50 and 200 µg/ml in infectious blood meals, SN-2 (**130**) reduced *Plasmodium* infection in mosquitoes by 90% and mean oocyst numbers by 60% [193].

Ferreira et al. [194] investigated the physiological effects of various plant-derived beta-glycosides and their aglycones on *Leishmania* spp. viability in female sand fly *Lutzomyia* *longipalpis* and in vitro culture. Oral administration of the toxic aglycone mandelonitrile (**131**) in sugar diets reduced infection prevalence and *L.* *mexicana* parasite numbers in sand fly guts, and both mandelonitrile (**131**) and esculetin (**132**) had strong anti-*Leishmania* activities in in vitro cell cultures [194].

Substituted anthraquinones inspired by potently active antischistosomial compounds isolated from *Hemerocallis* *fulva* (Asphodelaceae) roots were synthesized into anthraquinones A-S and evaluated for filaricidal activity against microfilariae and adult worms of *B.* *malayi*. From these derivatizations, anthraquinone K (**133**) exerted 100% worm mortality at 5 ppm in 3 days and caused marked distortions in intrauterine embryos [195]. In 2013, Yadav et al. isolated five diarylheptanoid compounds from the leaves of *Alnus* *nepalensis* (Betulaceae) and tested for their anti-filariasis activity. Their analyses led to the identification of two potentially active agents, alnus dimer (**134**) and (*5S*)-5-hydroxy-1-(4-hydroxyphenyl)-7-(3,4-dihydroxyphenyl)-3-heptanone (**135**), exhibiting both macrofilaricidal (IC_50_ 6.57–10.31 µg/ml) and microfilaricidal (IC_50_ 11.05–22.10 µg/ml) effects [196].

**Fig. 10:**
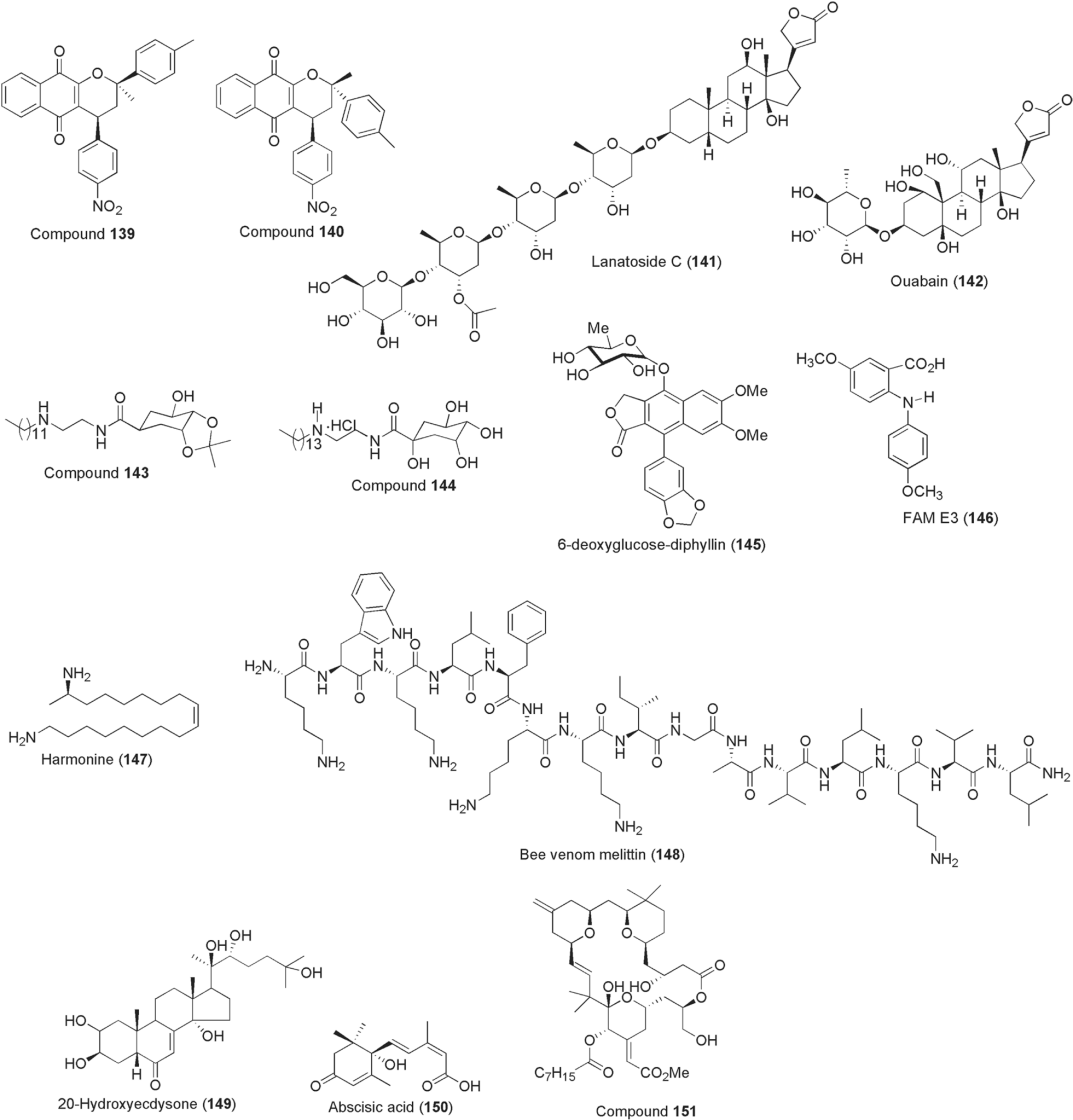
Chemical structures of compounds **139**–**151**. Diastereoisomers compound **139**, diastereoisomers compound **140**, lanatoside c (**141**), ouabain (**142**), quinic acid amide derivative **143**, quinic acid amide derivative **144**, diphyllin (**145**), anthranilic acid (fam e3) (**146**), (*17r,9z*)-1,17-diaminooctadec-9-ene (harmonine) (**147**), melittin (**148**), ecdysteroid (20e) (**149**), abscisic acid (**150**), compound **151**

Three acetylenic acids, octadeca-9,11,13-triynoic acid (**136**), (*13E*)-octadec-13-en-9,11-diynoic acid (**137**) and (*13E*)-octadec-13-en-11-ynoic acid (**138**), were isolated alongside other compounds from the leaves of a Madagascan plant, *Anacolosa* *pervilleana* (Olacaceae). The compounds **136**–**138** selectively inhibited the DENV RdRp (IC_50_s ~ 3 µM) at lower micromolar concentrations, were moderately potent against WNV RdRp and inactive against CHIKV [197]. Inspired by the structural framework of lapachol, a plant-derived naphthoquinone, several 1,4-pyran naphthoquinone derivatives were synthesized in the search for potential DENV inhibitors by da Costa et al. [198]. As a result, two diastereoisomers **139** and **140** (Fig. 10) potently inhibited DENV-2 replications in Vero cells achieving 99.0 and 99.6% blockade at IC_50_ values of 1.64 and 0.31 µM, respectively. Elsewhere, two independent drug repurposing studies have reported the antiviral activities of a number of FDA-approved plant-derived cardiac glycosides functionally acting through blockade of Na^+^/K^+^ ATPase pump channel. An earlier study screening a US drug collection library showed lanatoside C (**141**; derived from *Digitalis* *lanata*) exerted pan-DENV antiviral activity at an IC_50_ 0.19 µM in Huh-7 cells through inhibition of viral RNA and protein synthesis. However, the compound **141** weakly inhibits CHIKV by 38.66% at 1 µM [199]. More recently, Guo et al. screened two FDA-approved Na^+^/K^+^-ATPase inhibitors, digoxin and ouabain (**142**), against ZIKV under in vitro and in vivo conditions. Whilst both compounds displayed nanomolar IC_50_ anti-ZIKV inhibitory values, twice-potent ouabain (IC_50_ 48.39 nM) was found to block viral RNA synthesis by targeting Na^+^/K^+^-ATPase and reduced viral loads in mouse tissues [200]. In 2015, Zanello et al. synthesized a number of derivatives based on quinic acid backbone usually found in tomatoes, potatoes, coffee, carrots, etc. Quinic acid has previously been modified into potential antivirals and inspired synthesis of amide derivatives **143** and **144**, which inhibited replication of all DENV 1–4 serotypes at IC_50_ ≤ 10 µM [201].

Other reported anti-arboviral compounds include glycosylated diphyllin (**145**) and a diarylamine synthetic derivative of anthranilic acid (FAM E3; **146**). The former antiviral agent **145**, also known as patentiflorin A, is a naphthalene-derived compound isolated from *Justicia* *gendarussa* (Acanthaceae). Martinez-Lopez et al. reported its antiviral activity, demonstrating that the diphyllin component was the active principle against ZIKV and other flaviviruses, DENV, WNV, etc. Compound **145** potently blocked ZIKV infection (100% inhibition) at concentrations ~ 0.25–0.5 µM achieving an IC_50_ between 0.01 and 0.03 µM. The antiviral activity was mediated through prevention of endosomal acidification [202]. FAM E3 reduced up to 86% ZIKV replications and 96% infectivity at 3 µM during post-entry phase. The compound achieved an anti-ZIKV EC_50_ of 2.59 µM by binding to and stabilizing NS3 helicase [203].

### Miscellaneous sources

Apart from plants, insect gut microbiota and terrestrial microbes, exploration of other natural sources for bioactives has been reported especially from invertebrates. For instance, in 2012 Röhrich et al. identified (17R,9Z)-1,17-diaminooctadec-9-ene (harmonine; **147**) from the haemolymph of harlequin ladybird betele *Harmonia* *axyridis*. In addition to its activity against *Mycobacterium* *tuberculosis*, compound **147** reduced *P.* *falciparum* NF54 gametocytes at 4.8 µM to 18%, completely killing all gametocytes at 50 µM. Microgametogenesis was inhibited at IC_50_ value of 5.8 µM, reducing zygote formation to 17% and 1.2% at 4.8 and 50 µM, respectively. Offering harmonine at 10 µM in gametocytaemic blood meals to *Anopheles* *stephensi* mosquitoes resulted in 45/91 infected females with mean oocyst numbers of 1 [204].

A screen of 33 antimicrobial peptides from various sources, including toxins from bacteria, invertebrate stings and venoms, amphibian skin secretions, fish mucus and vertebrate antimicrobial peptides against *Plasmodium* sporogony in 2013 led to the identification of a bee killer effector peptide (melittin; **148**). At 50 µM, melittin consistently reduced *P.* *berghei* oocyst parasite prevalence by a mean 10% and intensity by 68% in mosquito midguts. Also the peptide reduced *P.* *falciparum* by an average of 57% [205]. Additional bee venom constituent, phospholipase A2, transgenically expressed in *An.* *stephensi* midguts effectively impaired *P.* *berghei* oocyst formation by 87% and blocked parasite transmissions to naïve mice [206]. Another potential source of bioactive peptides with *Plasmodium* transmission-blocking activity is from spiders. In this regard, an antimicrobial peptide gomesin isolated from haemocytes of spider *Acanthoscurria* *gomesiana* was reported. In addition to its antimalarial activity against asexual stages, the peptide at 50 µM inhibited *P.* *berghei* exflagellation of male gametes by 58% and formation of ookinetes by 100%. In mosquitoes the peptide reduced *Plasmodium* oocyst numbers without inducing noticeable toxicity effects [207].

Studies have implicated host cell lipid hijacking by vector-borne pathogens as a unifying strategy to complete their transmission life cycle and development (reviewed in [208]). But as a feasible intervention target to combat infectious transmissions, its translational applicability is still at infancy stages. In mosquitoes, for instance, lipid metabolism is controlled by pathways heavily dependent upon by developing sporogonic *Plasmodium* parasites, including the ecdysteroid (20E; **149**) hormone signalling [209, 210]. Topical application of steroid/non-steroid agonists, halofenozide, dibenzylhydrazines (e.g. methoxyfenozide) or microinjection of 20E itself into female *Anopheles* mosquitoes prior to infection manipulates steroid hormone titres, reducing susceptibility to *Plasmodium* [211–213]. In part, 20E manipulations boost basal innate immune responses that render mosquito midgut unfavourable for sporogonic parasite development.

The human-derived stress signalling isoprenoid molecule and phytohormone, abscisic acid (**150**), have a calcium signalling function essential in Apicomplexans [214, 215]. While compound **150** itself is inactive against gametocytes and asexual stages in vitro, Glennon et al. have demonstrated that either oral supplementation or pre-treatment of mature *Plasmodium* gametocytes reduces transmission to mosquitoes [216, 217]. In the mechanistic principle of its transmission-blocking, abscisic acid primes innate immune activation of infected hosts and appears to increase expression of mosquito nitric oxide synthase levels, in consequence mediating reduced infection prevalence in a nitric oxide- and NF-kB-dependent manner [216, 218].

Bryostatin 1 is a macrolide isolated from marine algae in 1982 and largely associated with pan-protein kinase C (PKC) modulation. While the compound itself was inactive against CHIKV (EC_50_ > 96 µM), structural simplification of the scaffold at A- and B-ring fragments gave **151** that outperformed other anti-CHIKV inhibitors mediating activity through PKC such as prostatin and reported jatrophanes (EC_50_ 0.8 µM). Compound **151** possessing C-8 gem-dimethyl + C-13-methylenyl substitutions exerted anti-CHIKV with an EC_50_ of 0.35 µM [219].

A summary of the discussed compounds has been provided in “Additional file 1”.

## Filling the gap in applicability

Leveraging of the existing and innovative approaches in delivery of the highlighted natural compounds is poised to expand their use beyond human treatments through disruption of pathogen transmission by insect vectors. Notably, the deployability of the suggested approaches could be crosscutting for control of various vector-borne diseases. Among these delivery mechanisms of transmission-blocking technologies would be by treatment of surfaces as reported by Paton et al. [220], where efficacious doses of atovaquone were delivered directly to the mosquito through contact exposure. Modelling studies also suggested that atovaquone would be effective when applied to long-lasting insecticide-treated nets, providing a potential route of administration for similar transmission-blocking chemistries.

Molecules can also be administered to animals as endectocides especially where less anthropogenic vectors such as *Anopheles* *arabiensis* mosquitoes are targeted. However, this approach will require drug properties that confer high residual activity, systemic activity and safety to both animals and humans such as topical and feed-through mosquito control systems using fipronil and ivermectin [221, 222]. Attractive sugar baits or other baited surfaces treated with transmission-blocking compounds may also provide a feasible route of administration as demonstrated in laboratory testing, semi-field and field application of disease vectors and harboured pathogens over the last decade [223–226]. For instance, the development of ivermectin-based attractive toxic sugar baits (ATSBs) in 10% sucrose solutions (0.01% ivermectin) against *An*. *arabiensis* resulted in > 95% mosquitoe knock-down 48 h post-feeding, suggesting its potential use for outdoor implementation [227].

Another route of administration of mosquito-stage drugs would be through nanoparticle formulations as sprays or biolarvicides, with engineered technology to ensure bioavailability of the drug in the adult mosquito. To our knowledge such robust technologies have not been developed yet and form a subject of further research. The use of adult vector artificial diets treated with the formulated natural compounds can also be explored to be deployed as auto-dissemination stations of the mosquito-stage transmission-blocking chemistries. Based on studies with electrostatic nettings for application of mosquito adulticides [228], another approach that we suggest is utilizing electrostatic dust treated with the drug and applied as wall lining, bed nets, electrostatic netting fitted eave-tubes or indoor wall sprays, which would pile on the adult mosquito body upon wing vibrations during flight.

Agonistic proteins aiding pathogen invasions and vector tissue colonization are potential candidates for drug targeting and transmission-blocking intervention designs. In view of this, small molecule mimetics were designed to target glycosaminoglycan anchorage of *Plasmodium*-impaired mosquito midgut invasions, reducing oocyst development by 99% [229, 230]. Other screening attempts have been subsequently directed against the *Anopheles* carboxypeptidase B [231]. Whether through enzymatic inhibition screens, polymeric conjugation with such exemplar proteins or nanoformulated as drug delivery carriers [232], the potentially active natural products could be applied as anchorage inhibitors and/or immune boosters of basal antipathogen responses. Also, advancements in synthetic biology and bioengineering [233] could be adopted for sustainable release and delivery of effector antipathogen products on trigger by pathogen-induced mechanisms during vector infection. This approach is best suited when the biosynthetic gene cluster(s) of a given bioactive natural compound are known and utilized to bioengineer the stable obligate microbiomes and viromes in a paratransgenic manner. This novelty in effector release could offer unprecedented routes of transmission-blocking interventions.

Besides that, natural products could be chemically modified by molecular hybridization with clinically approved chemistries for improved in vivo potencies and pharmacokinetics profiles.

## Conclusions

In this review, we have provided a list of natural compounds reported to have potential arthropod-borne pathogen transmission-blocking activities and a contemporary perspective on how such molecules could be integrated into design of control interventions. These molecules provide a blueprint towards (i) scaffold advancement to better drug-like pathogen transmission-blocking leads with improved therapeutic indices, (ii) spurring the continuous search for other potent compounds from various natural sources and (iii) providing a roadmap for translating the laboratory findings and innovatively lead to designing novel community-viable interventions that aid in reducing disease endemicity. For chemical groups that share similar activity, we propose that further chemical modelling is needed to establish structural scaffolds that can be used to inspire synthetic analogues of multiple modes of action to help combat one or more diseases. The provided strategies, in addition to other upcoming next-generation approaches, could be followed in focused design of sustainable delivery systems of these molecules towards acceleration for reduced disease spread amongst vulnerable human hosts. We however noted with mindful concern the lack of compounds investigated against the vector infective forms of *Leishmania* metacyclic promastigotes and trypanosome metacyclic trypomastigotes. Like other diseases of public health concern, there is need to address the existing gap through revitalized discovery efforts. We believe this review will inspire more discovery efforts within the field of natural products for development of vector-based approaches from the potent molecules endowed with pathogen transmission incapacitations.

## Supplementary Information

**Additional file 1. **Summarized details of the highlighted compounds 1 - 151: their chemical names, class, pathogens tested, and described mode of action.

## Data Availability

All datasets generated or analysed during this study are included in this published article.

## References

[1] Debebe Y, Hill SR, Birgersson G, Tekie H, Ignell R (2020). *Plasmodium*
*falciparum* gametocyte-induced volatiles enhance attraction of *Anopheles* mosquitoes in the field. Malar J.

[2] De Moraes CM, Wanjiku C, Stanczyk NM, Pulido H, Sims JW, Betz HS (2018). Volatile biomarkers of symptomatic and asymptomatic malaria infection in humans. Proc Natl Acad Sci.

[3] Busula AO, Bousema T, Mweresa CK, Masiga D, Logan JG, Sauerwein RW (2017). Gametocytemia and attractiveness of *Plasmodium*
*falciparum*–infected Kenyan children to *Anopheles*
*gambiae* mosquitoes. J Infect Dis.

[4] Batista EPA, Costa EFM, Silva AA (2014). Anopheles darlingi (Diptera: Culicidae) displays increased attractiveness to infected individuals with *Plasmodium*
*vivax* gametocytes. Parasit Vectors.

[5] da Tavares DS, Salgado VR, Miranda JC, Mesquita PRR, de Rodrigues FM, Barral-Netto M (2018). Attraction of phlebotomine sandflies to volatiles from skin odors of individuals residing in an endemic area of tegumentary leishmaniasis. PLoS ONE.

[6] Lequime S, Paul RE, Lambrechts L (2016). Determinants of arbovirus vertical transmission in mosquitoes. PLOS Pathog.

[7] Du S, Liu Y, Liu J, Zhao J, Champagne C, Tong L (2019). *Aedes* mosquitoes acquire and transmit Zika virus by breeding in contaminated aquatic environments. Nat Commun.

[8] Wei Xiang BW, Saron WAA, Stewart JC, Hain A, Walvekar V, Missé D (2022). Dengue virus infection modifies mosquito blood-feeding behavior to increase transmission to the host. Proc Natl Acad Sci.

[9] Maeno Y, Quang NT, Culleton R, Kawai S, Masuda G, Hori K (2017). Detection of the *Plasmodium*
*falciparum* Kelch-13 gene P553L mutation in sporozoites isolated from mosquito salivary glands in South-Central Vietnam. Parasit Vectors.

[10] Bell AS, Huijben S, Paaijmans KP, Sim DG, Chan BHK, Nelson WA (2012). Enhanced transmission of drug-resistant parasites to mosquitoes following drug treatment in rodent malaria. PLoS ONE.

[11] Van Bockstal L, Hendrickx S, Maes L, Caljon G (2020). Sand fly studies predict transmission potential of drug-resistant *Leishmania*. Trends Parasitol.

[12] Delang L, Yen P-S, Vallet T, Vazeille M, Vignuzzi M, Failloux A-B (2018). Differential transmission of antiviral drug-resistant chikungunya viruses by *Aedes* mosquitoes. MSphere Am Soc Microbiol.

[13] Schorderet-Weber S, Noack S, Selzer PM, Kaminsky R (2017). Blocking transmission of vector-borne diseases. Int J Parasitol Drugs Drug Resist.

[14] Gonçalves D, Hunziker P (2016). Transmission-blocking strategies: the roadmap from laboratory bench to the community. Malar J.

[15] Sinden RE (2017). Developing transmission-blocking strategies for malaria control. PLoS Pathog.

[16] Conway MJ, Colpitts TM, Fikrig E (2014). Role of the vector in arbovirus transmission. Annu Rev Virol Annu Rev.

[17] Leitner WW, Wali T, Kincaid R, Costero-Saint DA (2015). Arthropod vectors and disease transmission: translational aspects. PLoS Negl Trop Dis.

[18] Johnston KL, Hong WD, Turner JD, O’Neill PM, Ward SA, Taylor MJ (2021). Anti-*Wolbachia* drugs for filariasis. Trends Parasitol.

[19] Dong S, Dimopoulos G (2021). Antiviral compounds for blocking arboviral transmission in mosquitoes. Viruses.

[20] Torto B (2019). Innovative approaches to exploit host plant metabolites in malaria control. Pest Manag Sci.

[21] Delves MJ, Angrisano F, Blagborough AM (2018). Antimalarial transmission-blocking interventions: past, present, and future. Trends Parasitol.

[22] Birkholtz L-M, Coetzer TL, Mancama D, Leroy D, Alano P (2016). Discovering new transmission-blocking antimalarial compounds: challenges and opportunities. Trends Parasitol.

[23] Rodrigues T, Prudêncio M, Moreira R, Mota MM, Lopes F (2012). Targeting the liver stage of malaria parasites: a yet unmet goal. J Med Chem.

[24] Atanasov AG, Zotchev SB, Dirsch VM, Orhan IE, Banach M, Rollinger JM (2021). Natural products in drug discovery: advances and opportunities. Nat Rev Drug Discov.

[25] Newman DJ, Cragg GM (2020). Natural products as sources of new drugs over the nearly four decades from 01/1981 to 09/2019. J Nat Prod.

[26] Muema JM, Bargul JL, Njeru SN, Onyango JO, Imbahale SS (2017). Prospects for malaria control through manipulation of mosquito larval habitats and olfactory-mediated behavioural responses using plant-derived compounds. Parasit Vectors.

[27] Muturi EJ, Lagos-Kutz D, Dunlap C, Ramirez JL, Rooney AP, Hartman GL (2018). Mosquito microbiota cluster by host sampling location. Parasit Vectors.

[28] Gao H, Cui C, Wang L, Jacobs-Lorena M, Wang S (2020). Mosquito microbiota and implications for disease control. Trends Parasitol.

[29] Saraiva RG, Dimopoulos G (2020). Bacterial natural products in the fight against mosquito-transmitted tropical diseases. Nat Prod Rep.

[30] Lacerda AF, Pelegrini PB, de Oliveira DM, Vasconcelos ÉAR, Grossi-de-Sá MF (2016). Anti-parasitic peptides from arthropods and their application in drug therapy. Front Microbiol.

[31] Chernysh S, Kim SI, Bekker G, Pleskach VA, Filatova NA, Anikin VB (2002). Antiviral and antitumor peptides from insects. Proc Natl Acad Sci.

[32] Cirimotich CM, Dong Y, Clayton AM, Sandiford SL, Souza-Neto JA, Mulenga M (2011). Natural microbe-mediated refractoriness to *Plasmodium* infection in *Anopheles*
*gambiae*. Science.

[33] Ramirez JL, Short SM, Bahia AC, Saraiva RG, Dong Y, Kang S (2014). *Chromobacterium* Csp_P reduces malaria and dengue infection in vector mosquitoes and has entomopathogenic and *in*
*vitro* anti-pathogen activities. PLoS Pathog.

[34] Saraiva RG, Huitt-Roehl CR, Tripathi A, Cheng Y-Q, Bosch J, Townsend CA (2018). *Chromobacterium* spp. mediate their anti-*Plasmodium* activity through secretion of the histone deacetylase inhibitor romidepsin. Sci Rep.

[35] Saraiva RG, Fang J, Kang S, Angleró-Rodríguez YI, Dong Y, Dimopoulos G (2018). Aminopeptidase secreted by *Chromobacterium* sp. Panama inhibits dengue virus infection by degrading the E protein. PLoS Negl Trop Dis..

[36] Short SM, van Tol S, MacLeod HJ, Dimopoulos G (2018). Hydrogen cyanide produced by the soil bacterium *Chromobacterium* sp. Panama contributes to mortality in *Anopheles*
*gambiae* mosquito larvae. Sci Rep.

[37] Tavella TA, da Silva NSM, Spillman N, Kayano ACAV, Cassiano GC, Vasconcelos AA (2021). Violacein-induced chaperone system collapse underlies multistage antiplasmodial activity. ACS Infect Dis.

[38] Nattoh G, Maina T, Makhulu EE, Mbaisi L, Mararo E, Otieno FG, et al. Horizontal transmission of the symbiont Microsporidia MB in *Anopheles**arabiensis*. Front Microbiol. 2021;12. 10.3389/fmicb.2021.647183 PMC8355901 34394019

[39] Herren JK, Mbaisi L, Mararo E, Makhulu EE, Mobegi VA, Butungi H (2020). A microsporidian impairs *Plasmodium*
*falciparum* transmission in *Anopheles*
*arabiensis* mosquitoes. Nat Commun.

[40] Gonzalez-Ceron L, Santillan F, Rodriguez MH, Mendez D, Hernandez-Avila JE (2003). Bacteria in midguts of field-collected *Anopheles*
*albimanus* block *Plasmodium*
*vivax* sporogonic development. J Med Entomol.

[41] Steyn A, Roets F, Botha A (2016). Yeasts associated with *Culex*
*pipiens* and *Culex*
*theileri* mosquito larvae and the effect of selected yeast strains on the ontogeny of *Culex*
*pipiens*. Microb Ecol.

[42] Cappelli A, Ulissi U, Valzano M, Damiani C, Epis S, Gabrielli MG (2014). A *Wickerhamomyces*
*anomalus* killer strain in the malaria vector *Anopheles*
*stephensi*. PLoS ONE.

[43] Ricci I, Damiani C, Scuppa P, Mosca M, Crotti E, Rossi P (2011). The yeast *Wickerhamomyces*
*anomalus* (Pichia anomala) inhabits the midgut and reproductive system of the Asian malaria vector *Anopheles*
*stephensi*. Env Microbiol.

[44] Cappelli A, Valzano M, Cecarini V, Bozic J, Rossi P, Mensah P (2019). Killer yeasts exert anti-plasmodial activities against the malaria parasite *Plasmodium*
*berghei* in the vector mosquito *Anopheles*
*stephensi* and in mice. Parasit Vectors.

[45] Valzano M, Cecarini V, Cappelli A, Capone A, Bozic J, Cuccioloni M. A yeast strain associated to *Anopheles* mosquitoes produces a toxin able to kill malaria parasites. Malar J. 2016;15. 10.1186/s12936-015-1059-7 PMC4709964 26754943

[46] Czesny B, Goshu S, Cook JL, Williamson KC (2009). The proteasome inhibitor Epoxomicin has potent *Plasmodium*
*falciparum* gametocytocidal activity. Antimicrob Agents Chemother.

[47] Aminake MN, Schoof S, Sologub L, Leubner M, Kirschner M, Arndt H (2011). Thiostrepton and derivatives exhibit antimalarial and gametocytocidal activity by dually targeting parasite. Antimicrob Agents Chemother.

[48] Campell CW, Fisher MH, Stapley EO, Albers-Schonberg G, Jacob TA (1983). Ivermectin: a potent new antiparasitic agent. Science.

[49] Pinilla YT, Lopes CP, Sampaio VS, Andrade FS, Melo GC, Orfanó AS (2018). Promising approach to reducing Malaria transmission by ivermectin: Sporontocidal effect against *Plasmodium*
*vivax* in the South American vectors *Anopheles*
*aquasalis* and *Anopheles*
*darlingi*. PLoS Negl Trop Dis..

[50] Kobylinski KC, Escobedo-Vargas KS, López-Sifuentes VM, Durand S, Smith ES, Baldeviano GC (2017). Ivermectin susceptibility, sporontocidal effect, and inhibition of time to re-feed in the Amazonian malaria vector *Anopheles*
*darlingi*. Malar J.

[51] Mendes AM, Albuquerque IS, Machado M, Pissarra J, Meireles P, Prudêncio M (2017). Inhibition of *Plasmodium* liver infection by ivermectin. Antimicrob Agents Chemother.

[52] Kobylinski KC, Foy BD, Richardson JH (2012). Ivermectin inhibits the sporogony of *Plasmodium*
*falciparum* in *Anopheles*
*gambiae*. Malar J.

[53] de Sampaio VS, da Rivas GBS, Kobylinski K, Pinilla YT, Pimenta PFP, Lima JBP (2017). What does not kill it makes it weaker: effects of sub-lethal concentrations of ivermectin on the locomotor activity of *Anopheles*
*aquasalis*. Parasit Vectors..

[54] Lyimo IN, Kessy ST, Mbina KF, Daraja AA, Mnyone LL (2017). Ivermectin-treated cattle reduces blood digestion, egg production and survival of a free-living population of *Anopheles*
*arabiensis* under semi-field condition in south-eastern Tanzania. Malar J.

[55] Pooda HS, Rayaisse J-B, de Hien DFS, Lefèvre T, Yerbanga SR, Bengaly Z (2015). Administration of ivermectin to peridomestic cattle: a promising approach to target the residual transmission of human malaria. Malar J.

[56] Pooda SH, Mouline K, De Meeûs T, Bengaly Z, Solano P (2013). Decrease in survival and fecundity of *Glossina*
*palpalis* gambiensis vanderplank 1949 (Diptera: Glossinidae) fed on cattle treated with single doses of ivermectin. Parasit Vectors.

[57] Kobylinski KC, Deus KM, Butters MP, Hongyu T, Gray M, da Silva IM (2010). The effect of oral anthelmintics on the survivorship and re-feeding frequency of anthropophilic mosquito disease vectors. Acta Trop.

[58] Held J, Gebru T, Kalesse M, Jansen R, Gerth K, Müller R (2014). Antimalarial activity of the myxobacterial macrolide chlorotonil A. Antimicrob Agents Chemother.

[59] Pastrana-mena R, Mathias DK, Delves M, Rajaram K, King JG, Yee R (2016). A malaria transmission-blocking (+)-Usnic acid derivative prevents *Plasmodium* zygote-to-ookinete maturation in the mosquito midgut. ACS Chem Biol.

[60] Lauinger IL, Vivas L, Perozzo R, Stairiker C, Tarun A, Zloh M (2013). Potential of lichen secondary metabolites against *Plasmodium* liver stage parasites with FAS-II as the potential target. J Nat Prod.

[61] Zhang G, Niu G, Franca CM, Dong Y, Wang X, Butler NS (2015). *Anopheles* midgut FREP1 mediates *Plasmodium* invasion. J Biol Chem.

[62] Niu G, Wang B, Zhang G, King JB, Cichewicz RH, Li J (2015). Targeting mosquito FREP1 with a fungal metabolite blocks malaria transmission. Sci Rep.

[63] Niu G, Wang X, Hao Y, Kandel S, Niu G, Raptis RG (2021). A novel fungal metabolite inhibits *Plasmodium*
*falciparum* transmission and infection. Parasit Vectors.

[64] Niu G, Hao Y, Wang X, Gao J-M, Li J (2020). Fungal metabolite Asperaculane B inhibits malaria infection and transmission. Molecules.

[65] Douglas RG, Reinig M, Neale M, Frischknecht F (2018). Screening for potential prophylactics targeting sporozoite motility through the skin. Malar J.

[66] Sun W, Tanaka TQ, Magle CT, Huang W, Southall N, Huang R (2014). Chemical signatures and new drug targets for gametocytocidal drug development. Sci Rep.

[67] Maron MI, Magle CT, Czesny B, Turturice BA, Huang R, Zheng W (2015). Maduramicin rapidly eliminates malaria parasites and potentiates the gametocytocidal activity of the pyrazoleamide PA21A050. Antimicrob Agents Chemother.

[68] D’Alessandro S, Corbett Y, Ilboudo DP, Misiano P, Dahiya N, Abay SM (2015). Salinomycin and other ionophores as a new class of antimalarial drugs with transmission-blocking activity. Antimicrob Agents Chemother.

[69] Derbyshire ER, Prudêncio M, Mota MM, Clardy J (2012). Liver-stage malaria parasites vulnerable to diverse chemical scaffolds. Proc Natl Acad Sci.

[70] van Pelt-Koops JC, Pett HE, Graumans W, van der Vegte-Bolmer M, van Gemert GJ, Rottmann M (2012). The spiroindolone drug candidate NITD609 potently inhibits gametocytogenesis and blocks *Plasmodium*
*falciparum* transmission to anopheles mosquito vector. Antimicrob Agents Chemother.

[71] Rottmann M, McNamara C, Yeung BKS, Lee MCS, Zou B, Russell B (2010). Spiroindolones, a potent compound class for the treatment of malaria. Science.

[72] Schiefer A, Hübner MP, Krome A, Lämmer C, Ehrens A, Aden T (2020). Corallopyronin A for short-course anti-wolbachial, macrofilaricidal treatment of filarial infections. PLoS Negl Trop Dis.

[73] Schiefer A, Schmitz A, Schäberle TF, Specht S, Lämmer C, Johnston KL (2012). Corallopyronin A specifically targets and depletes essential obligate *Wolbachia* endobacteria from filarial nematodes in vivo. J Infect Dis.

[74] Xu Z, Fang S-M, Bakowski MA, Rateb ME, Yang D, Zhu X (2019). Discovery of kirromycins with anti-Wolbachia activity from *Streptomyces* sp. CB00686. ACS Chem Biol.

[75] von Geldern TW, Morton HE, Clark RF, Brown BS, Johnston KL, Ford L (2019). Discovery of ABBV-4083, a novel analog of Tylosin A that has potent anti-*Wolbachia* and anti-filarial activity. PLoS Negl Trop Dis.

[76] Jacobs RT, Lunde CS, Freund YR, Hernandez V, Li X, Xia Y (2019). Boron-pleuromutilins as anti-*Wolbachia* agents with potential for treatment of onchocerciasis and lymphatic filariasis. J Med Chem.

[77] Inukai M, Nakajima M, Osawa M, Haneishi T, Arai M (1978). Globomycin, a new peptide antibiotic with spheroplast-forming activity. II. Isolation and physico-chemical and biological characterization. J Antibiot (Tokyo)..

[78] Johnston KL, Wu B, Guimarães A, Ford L, Slatko BE, Taylor MJ (2010). Lipoprotein biosynthesis as a target for anti-*Wolbachia* treatment of filarial nematodes. Parasit Vectors.

[79] Rao R, Weil GJ (2002). *In*
*vitro* effects of antibiotics on *Brugia*
*malayi* worm survival and reproduction. J Parasitol.

[80] Bulman CA, Chappell L, Gunderson E, Vogel I, Beerntsen B, Slatko BE (2021). The Eagle effect in the *Wolbachia*-worm symbiosis. Parasit Vectors.

[81] Rateb ME, Yang D, Vodanovic-Jankovic S, Yu Z, Kron MA, Shen B (2015). Adipostatins A-D from *Streptomyces* sp. 4875 inhibiting *Brugia*
*malayi* asparaginyl-tRNA synthetase and killing adult *Brugia*
*malayi* parasites. J Antibiot (Tokyo)..

[82] Yu Z, Vodanovic-Jankovic S, Kron M, Shen B (2012). New WS9326A congeners from *Streptomyces* sp. 9078 inhibiting *Brugia*
*malayi* asparaginyl-tRNA synthetase. Org Lett.

[83] Yu Z, Vodanovic-Jankovic S, Ledeboer N, Huang S-X, Rajski SR, Kron M (2011). Tirandamycins from *Streptomyces* sp. 17944 inhibiting the parasite *Brugia*
*malayi* asparagine tRNA synthetase. Org Lett.

[84] Rausch K, Hackett BA, Weinbren NL, Reeder SM, Sadovsky Y, Hunter CA (2017). Screening bioactives reveals nanchangmycin as a broad spectrum antiviral active against zika virus. Cell Rep.

[85] Estoppey D, Lee CM, Janoschke M, Lee BH, Wan KF, Dong H (2017). The natural product cavinafungin selectively interferes with zika and dengue virus replication by inhibition of the host signal peptidase. Cell Rep.

[86] Rox K, Heyner M, Krull J, Harmrolfs K, Rinne V, Hokkanen J (2021). Physiologically based pharmacokinetic/pharmacodynamic model for the treatment of dengue infections applied to the broad spectrum antiviral Soraphen A. ACS Pharmacol Transl Sci.

[87] Hans P, Katharina R, Suryanarayana BNV, Loreen W, Sven-Kevin H, Philipp K (2021). Labyrinthopeptins exert broad-spectrum antiviral activity through lipid-binding-mediated virolysis. J Virol.

[88] Barrows NJ, Campos RK, Powell ST, Prasanth KR, Schott-Lerner G, Soto-Acosta R (2016). A screen of FDA-approved drugs for inhibitors of zika virus infection. Cell Host Microbe.

[89] Mastrangelo E, Pezzullo M, De Burghgraeve T, Kaptein S, Pastorino B, Dallmeier K (2012). Ivermectin is a potent inhibitor of flavivirus replication specifically targeting NS3 helicase activity: new prospects for an old drug. J Antimicrob Chemother.

[90] Varghese FS, Kaukinen P, Gläsker S, Bespalov M, Hanski L, Wennerberg K (2016). Discovery of berberine, abamectin and ivermectin as antivirals against chikungunya and other alphaviruses. Antiviral Res.

[91] Diamond MS, Zachariah M, Harris E (2002). Mycophenolic Acid inhibits dengue virus infection by preventing replication of viral RNA. Virology.

[92] Min Q, Feng Y, Bo Z, Gang Z, Robida JM, Zhiming Y (2009). Cyclosporine inhibits flavivirus replication through blocking the interaction between host cyclophilins and viral NS5 protein. Antimicrob Agents Chemother..

[93] Dong S, Kang S, Dimopoulos G (2019). Identification of anti-flaviviral drugs with mosquitocidal and anti-Zika virus activity in *Aedes*
*aegypti*. PLoS Negl Trop Dis.

[94] Raveh A, Delekta PC, Dobry CJ, Peng W, Schultz PJ, Blakely PK (2013). Discovery of potent broad spectrum antivirals derived from marine actinobacteria. PLoS ONE.

[95] Raekiansyah M, Mori M, Nonaka K, Agoh M, Shiomi K, Matsumoto A (2017). Identification of novel antiviral of fungus-derived brefeldin A against dengue viruses. Trop Med Health.

[96] Chunfeng L, Shulong Z, Yong-Qiang D, Dapei L, Kislay P, Natalie Q (2021). Azithromycin protects against zika virus infection by upregulating virus-induced type I and III interferon responses. Antimicrob Agents Chemother.

[97] Rothan HA, Bahrani H, Mohamed Z, Teoh TC, Shankar EM, Rahman NA (2015). A combination of doxycycline and ribavirin alleviated chikungunya infection. PLoS ONE.

[98] Gupta DK, Kaur P, Leong ST, Tan LT, Prinsep MR, Chu JJ (2014). Anti-chikungunya viral activities of Aplysiatoxin-related compounds from the marine cyanobacterium *Trichodesmium*
*erythraeum*. Mar Drugs.

[99] Kang S, Shields AR, Jupatanakul N, Dimopoulos G (2014). Suppressing dengue-2 infection by chemical inhibition of *Aedes*
*aegypti* host factors. PLoS Negl Trop Dis.

[100] Nyasembe VO, Tchouassi DP, Pirk CWW, Sole CL, Torto B (2018). Host plant forensics and olfactory-based detection in Afro-tropical mosquito disease vectors. PLoS Negl Trop Dis.

[101] Nyasembe VO, Peter EA, Sawa P, Tumlinson JH, Borgemeister C, Torto B (2014). *Plasmodium*
*falciparum* infection increases *Anopheles*
*gambiae* attraction to nectar sources and sugar uptake. Curr Biol..

[102] Agha SB, Alvarez M, Becker M, Fèvre EM, Junglen S, Borgemeister C (2020). Invasive alien plants in Africa and the potential emergence of mosquito-borne arboviral diseases—a review and research outlook. Viruses.

[103] Wanjiku C, Tchouassi DP, Sole CL, Pirk C, Torto B (2021). Plant sugar feeding patterns of wild-caught *Aedes*
*aegypti* from dengue endemic and non-endemic areas of Kenya. Med Vet Entomol.

[104] Hassaballa IB, Sole CL, Cheseto X, Torto B, Tchouassi DP (2021). Afrotropical sand fly-host plant relationships in a leishmaniasis endemic area. Kenya PLoS Negl Trop Dis.

[105] Abbasi I, TrancosoLopo de Queiroz A, Kirstein OD, Nasereddin A, Horwitz BZ, Hailu A (2018). Plant-feeding phlebotomine sand flies, vectors of leishmaniasis, prefer *Cannabis*
*sativa*. Proc Natl Acad Sci..

[106] Stone CM, Witt ABR, Walsh GC, Foster WA, Murphy ST (2018). Would the control of invasive alien plants reduce malaria transmission? A review. Parasit Vectors.

[107] Muller GC, Junnila A, Traore MM, Traore SF, Doumbia S, Sissoko F (2017). The invasive shrub *Prosopis*
*juliflora* enhances the malaria parasite transmission capacity of *Anopheles* mosquitoes: a habitat manipulation experiment. Malar J.

[108] Díaz-Albiter HM, Ferreira TN, Costa SG, Rivas GB, Gumiel M, Cavalcante DR (2016). Everybody loves sugar: first report of plant feeding in triatomines. Parasit Vectors.

[109] Nyasembe VO, Cheseto X, Kaplan F, Foster WA, Teal EA, Tumlinson JH (2015). The invasive American weed *Parthenium*
*hysterophorus* can negatively impact malaria control in Africa. PLoS ONE.

[110] Sissoko F, Junnila A, Traore MM, Traore SF, Doumbia S, Dembele SM (2019). Frequent sugar feeding behavior by *Aedes*
*aegypti* in Bamako, Mali makes them ideal candidates for control with attractive toxic sugar baits (ATSB). PLoS ONE.

[111] Müller GC, Beier JC, Traore SF, Toure MB, Traore MM, Bah S (2010). Field experiments of *Anopheles*
*gambiae* attraction to local fruits/seedpods and flowering plants in Mali to optimize strategies for malaria vector control in Africa using attractive toxic sugar bait methods. Malar J.

[112] Manda H, Gouagna LC, Foster WA, Jackson RR, Beier JC, Githure JI (2007). Effect of discriminative plant-sugar feeding on the survival and fecundity of *Anopheles*
*gambiae*. Malar J.

[113] Hien DFDS, Paré PSL, Cooper A, Koama BK, Guissou E, Yaméogo KB (2021). Contrasting effects of the alkaloid ricinine on the capacity of *Anopheles*
*gambiae* and *Anopheles*
*coluzzii* to transmit *Plasmodium*
*falciparum*. Parasit Vectors.

[114] Hien DFS, Dabiré KR, Roche B, Diabaté A, Yerbanga RS, Cohuet A (2016). Plant-mediated effects on mosquito capacity to transmit human malaria. PLOS Pathog..

[115] Almire F, Terhzaz S, Terry S, McFarlane M, Gestuveo RJ, Szemiel AM (2021). Sugar feeding protects against arboviral infection by enhancing gut immunity in the mosquito vector *Aedes*
*aegypti*. PLOS Pathog.

[116] Gualtieri MJ, Malafronte N, Vassallo A, Braca A, Cotugno R, Vasaturo M (2014). Bioactive limonoids from the leaves of *Azaridachta*
*indica* (Neem). J Nat Prod.

[117] Chianese G, Yerbanga SR, Lucantoni L, Habluetzel A, Basilico N, Taramelli D (2010). Antiplasmodial triterpenoids from the fruits of Neem, *Azadirachta*
*indica*. J Nat Prod.

[118] Billker O, Shaw MK, Jones IANW, Ley SV, Luntz AJM, Sinden RE (2002). Azadirachtin disrupts formation of organised microtubule arrays during microgametogenesis of *Plasmodium*
*berghei*. J Eukaryot Microbiol..

[119] Tapanelli S, Chianese G, Lucantoni L, Yerbanga RS, Habluetzel A, Taglialatela-Scafati O (2016). Transmission blocking effects of neem (*Azadirachta*
*indica*) seed kernel limonoids on *Plasmodium*
*berghei* early sporogonic development. Fitoterapia.

[120] Dahiya N, Chianese G, Abay SM, Taglialatela-Scafati O, Esposito F, Lupidi G (2016). In vitro and ex vivo activity of an *Azadirachta*
*indica* A.Juss. seed kernel extract on early sporogonic development of *Plasmodium* in comparison with azadirachtin A, its most abundant constituent. Phytomedicine.

[121] Lucantoni L, Yerbanga RS, Lupidi G, Pasqualini L, Esposito F, Habluetzel A (2010). Transmission blocking activity of a standardized neem (*Azadirachta*
*indica*) seed extract on the rodent malaria parasite *Plasmodium*
*berghei* in its vector *Anopheles*
*stephensi*. Malar J.

[122] Abay SM, Lucantoni L, Dahiya N, Dori G, Dembo EG, Esposito F (2015). *Plasmodium* transmission blocking activities of *Vernonia*
*amygdalina* extracts and isolated compounds. Malar J.

[123] Sirignano C, Hammami S, El Mokni R, Blagborough AM, Luciano P, Rigano D (2021). Polyoxygenated germacranes from *Daucus*
*carota* and their antimalarial transmission blocking activity. Phytochemistry.

[124] Sirignano C, Snene A, Tenoh AR, El Mokni R, Rigano D, Habluetzel A (2019). Daucovirgolides I-L, four congeners of the antimalarial daucovirgolide G from *Daucus*
*virgatus*. Fitoterapia.

[125] Sirignano C, Snene A, Rigano D, Tapanelli S, Formisano C, Luciano P (2017). Angeloylated germacranolides from *Daucus*
*virgatus* and their *Plasmodium* transmission blocking activity. J Nat Prod.

[126] Balaich JN, Mathias DK, Torto B, Jackson BT, Tao D, Ebrahimi B (2016). The nonartemisinin sesquiterpene lactones parthenin and parthenolide block *Plasmodium*
*falciparum* sexual stage transmission. Antimicrob Agents Chemother.

[127] Moyo P, Kunyane P, Selepe MA, Eloff JN, Niemand J, Louw AI (2019). Bioassay-guided isolation and identification of gametocytocidal compounds from *Artemisia*
*afra* (Asteraceae). Malar J.

[128] Lelièvre J, Almela MJ, Lozano S, Miguel C, Franco V, Leroy D (2012). Activity of clinically relevant antimalarial drugs on *Plasmodium*
*falciparum* mature gametocytes in an ATP bioluminescence “Transmission Blocking” assay. PLoS ONE.

[129] Witmer K, Dahalan FA, Delves MJ, Yahiya S, Watson OJ, Straschil U (2020). Transmission of artemisinin-resistant malaria parasites to mosquitoes under antimalarial drug pressure. Antimicrob Agents Chemother..

[130] Beshir KB, Sutherland CJ, Sawa P, Drakeley CJ, Okell L, Mweresa CK (2013). Residual *Plasmodium*
*falciparum* parasitemia in Kenyan children after artemisinin-combination therapy is associated with increased transmission to mosquitoes and parasite recurrence. J Infect Dis.

[131] Coertzen D, Reader J, Van Der Watt M, Nondaba SH, Gibhard L, Wiesner L (2018). Artemisone and artemiside are potent panreactive antimalarial agents that also synergize redox imbalance in *Plasmodium*
*falciparum* transmissible gametocyte stages. Antimicrob Agents Chemother.

[132] Wong HN, Padín-Irizarry V, Van der Watt ME, Reader J, Liebenberg W, Wiesner L, et al. Optimal 10-Aminoartemisinins with potent transmission-blocking capabilities for new artemisinin combination therapies activities against blood stage *P.**falciparum* including PfKI3 C580Y mutants and liver stage *P.**berghei* parasites. Front Chem. 2020; 901. 10.3389/fchem.2019.00901 PMC6967409 31998692

[133] Allard P-M, Leyssen P, Martin M-T, Bourjot M, Dumontet V, Eydoux C (2012). Antiviral chlorinated daphnane diterpenoid orthoesters from the bark and wood of *Trigonostemon*
*cherrieri*. Phytochemistry.

[134] Bourjot M, Delang L, Nguyen VH, Neyts J, Guéritte F, Leyssen P (2012). Prostratin and 12-*O*-tetradecanoylphorbol 13-acetate are potent and selective inhibitors of chikungunya virus replication. J Nat Prod.

[135] Bourjot M, Leyssen P, Neyts J, Dumontet V, Litaudon M, Trigocherrierin A (2014). a potent inhibitor of chikungunya virus replication. Molecules.

[136] Corlay N, Delang L, Girard-Valenciennes E, Neyts J, Clerc P, Smadja J (2014). Tigliane diterpenes from *Croton*
*mauritianus* as inhibitors of chikungunya virus replication. Fitoterapia.

[137] Nothias-Scaglia L-F, Retailleau P, Paolini J, Pannecouque C, Neyts J, Dumontet V (2014). Jatrophane diterpenes as inhibitors of chikungunya virus replication: Structure–activity relationship and discovery of a potent lead. J Nat Prod.

[138] Nothias-Scaglia L-F, Pannecouque C, Renucci F, Delang L, Neyts J, Roussi F (2015). Antiviral activity of diterpene esters on chikungunya virus and HIV replication. J Nat Prod.

[139] Techer S, Girard-Valenciennes E, Retailleau P, Neyts J, Guéritte F, Leyssen P (2015). Tonantzitlolones from *Stillingia*
*lineata* ssp. lineata as potential inhibitors of chikungunya virus. Phytochem Lett..

[140] Olivon F, Palenzuela H, Girard-Valenciennes E, Neyts J, Pannecouque C, Roussi F (2015). Antiviral activity of flexibilane and tigliane diterpenoids from *Stillingia*
*lineata*. J Nat Prod.

[141] Tan YP, Houston SD, Modhiran N, Savchenko AI, Boyle GM, Young PR (2019). Stachyonic acid: a dengue virus inhibitor from *Basilicum*
*polystachyon*. Chem A Eur J..

[142] Baltina LA, Tasi Y-T, Huang S-H, Lai H-C, Baltina LA, Petrova SF (2019). Glycyrrhizic acid derivatives as Dengue virus inhibitors. Bioorg Med Chem Lett.

[143] Baltina LA, Lai H-C, Liu Y-C, Huang S-H, Hour M-J, Baltina LA (2021). Glycyrrhetinic acid derivatives as Zika virus inhibitors: synthesis and antiviral activity *in*
*vitro*. Bioorg Med Chem.

[144] Cirne-Santos CC, Souza barros de C, Oliveira de MC, Rabelo VW-H, Azevedo RC, Teixeira VL (2020). In vitro studies on the inhibition of replication of Zika and Chikungunya viruses by dolastane isolated from seaweed *Canistrocarpus*
*cervicornis*. Sci Rep.

[145] Loe MWC, Hao E, Chen M, Li C, Lee RCH, Zhu IXY (2020). Betulinic acid exhibits antiviral effects against dengue virus infection. Antiviral Res.

[146] Misra S, Verma M, Mishra SK, Srivastava S, Lakshmi V, Misra-Bhattacharya S (2011). Gedunin and photogedunin of *Xylocarpus*
*granatum* possess antifilarial activity against human lymphatic filarial parasite *Brugia*
*malayi* in experimental rodent host. Parasitol Res.

[147] Kalani K, Kushwaha V, Verma R, Murthy PK, Srivastava SK (2013). Glycyrrhetinic acid and its analogs: a new class of antifilarial agents. Bioorg Med Chem Lett.

[148] Saini P, Gayen P, Kumar D, Nayak A, Mukherjee N, Mukherjee S (2014). Antifilarial effect of ursolic acid from *Nyctanthes*
*arbortristis*: molecular and biochemical evidences. Parasitol Int.

[149] Kushwaha V, Saxena K, Verma R, Verma SK, Katoch D, Kumar N (2016). Antifilarial activity of diterpenoids from *Taxodium*
*distichum*. Parasit Vectors.

[150] Peatey CL, Leroy D, Gardiner DL, Trenholme KR (2012). Anti-malarial drugs: how effective are they against *Plasmodium*
*falciparum* gametocytes?. Malar J.

[151] Achan J, Talisuna AO, Erhart A, Yeka A, Tibenderana JK, Baliraine FN (2011). Quinine, an old anti-malarial drug in a modern world: role in the treatment of malaria. Malar J.

[152] Chotivanich K, Sattabongkot J, Udomsangpetch R, Looareesuwan S, Day NPJ, Coleman RE (2006). Transmission-blocking activities of Quinine, Primaquine, and Artesunate. Antimicrob Agents Chemother.

[153] Vu H, Roullier C, Campitelli M, Trenholme KR, Gardiner DL, Andrews KT (2013). *Plasmodium* gametocyte inhibition identified from a natural-product-based fragment library. ACS Chem Biol.

[154] Forkuo AD, Ansah C, Mensah KB, Annan K, Gyan B, Theron A (2017). *In*
*vitro* anti-malarial interaction and gametocytocidal activity of cryptolepine. Malar J.

[155] Onambele LA, Riepl H, Fischer R, Pradel G, Prokop A, Aminake MN (2015). Synthesis and evaluation of the antiplasmodial activity of tryptanthrin derivatives. Int J Parasitol Drugs drug Resist.

[156] Goodman CD, Austarheim I, Mollard V, Mikolo B, Malterud KE, Mcfadden GI (2016). Natural products from *Zanthoxylum*
*heitzii* with potent activity against the malaria parasite. Malar J.

[157] Moyo P, Shamburger W, van der Watt ME, Reader J, de Sousa ACC, Egan TJ (2020). Naphthylisoquinoline alkaloids, validated as hit multistage antiplasmodial natural products. Int J Parasitol Drugs Drug Resist.

[158] Priyanka P, Burusco KK, Muna A, Holly M, Andrey G, Elena F-Á (2021). Lead optimization of dehydroemetine for repositioned use in malaria. Antimicrob Agents Chemother..

[159] Muema JM, Bargul JL, Mutunga JM, Obonyo MA, Mwakubambanya RS. Process of blocking *Plasmodium* gametocytogenesis and transmission using juliprosopine from *Prosopis**juliflora*. Kenya Intellectual Property Institute; 2020; KE/P/2020/3643.

[160] Carraz M, Jossang A, Franetich J-F, Siau A, Ciceron L, Hannoun L (2006). A plant-derived morphinan as a novel lead compound active against malaria liver stages. PLOS Med.

[161] Carraz M, Jossang A, Rasoanaivo P, Mazier D, Frappier F (2008). Isolation and antimalarial activity of new morphinan alkaloids on *Plasmodium*
*yoelii* liver stage. Bioorg Med Chem.

[162] de Lamballerie X, Ninove L, Charrel RN (2009). Antiviral treatment of chikungunya virus infection. Infect Disord Targets.

[163] Varghese SF, Bastian T, Naqiah AS, Diane S, Kai R, Nyman TA (2021). The antiviral alkaloid berberine reduces chikungunya virus-induced mitogen-activated protein kinase signaling. J Virol..

[164] Wan JJ, Brown RS, Margaret K (2021). Berberine chloride is an alphavirus inhibitor that targets nucleocapsid assembly. MBio.

[165] Parveen K, Meerra T, Hua LRC, Huixin C, Caiyun CK, Lee NM (2013). Inhibition of chikungunya virus replication by harringtonine, a novel antiviral that suppresses viral protein expression. Antimicrob Agents Chemother.

[166] Hwang J, Jiang A, Fikrig E (2019). A potent prolyl tRNA synthetase inhibitor antagonizes Chikungunya and Dengue viruses. Antiviral Res.

[167] Troost B, Mulder LM, Diosa-Toro M, van de Pol D, Rodenhuis-Zybert IA, Smit JM (2020). Tomatidine, a natural steroidal alkaloid shows antiviral activity towards chikungunya virus in vitro. Sci Rep.

[168] Diosa-Toro M, Troost B, van de Pol D, Heberle AM, Urcuqui-Inchima S, Thedieck K (2019). Tomatidine, a novel antiviral compound towards dengue virus. Antiviral Res.

[169] Kevin W, Pierson CT, Brian G, Kelly L, Michael E, Yi Z (2005). Castanospermine, a potent inhibitor of dengue virus infection *in*
*vitro* and *in*
*vivo*. J Virol..

[170] Wang P, Li L-F, Wang Q-Y, Shang L-Q, Shi P-Y, Yin Z (2014). Anti-dengue-virus activity and structure–activity relationship studies of lycorine derivatives. ChemMedChem.

[171] Chen H, Lao Z, Xu J, Li Z, Long H, Li D (2020). Antiviral activity of lycorine against Zika virus *in*
*vivo* and *in*
*vitro*. Virology.

[172] Zou G, Puig-Basagoiti F, Zhang B, Qing M, Chen L, Pankiewicz KW (2009). A single-amino acid substitution in West Nile virus 2K peptide between NS4A and NS4B confers resistance to lycorine, a flavivirus inhibitor. Virology.

[173] Ka S, Merindol N, Sow AA, Singh A, Landelouci K, Plourde MB (2021). Amaryllidaceae alkaloid cherylline inhibits the replication of dengue and zika viruses. Antimicrob Agents Chemother.

[174] Yang S, Xu M, Lee EM, Gorshkov K, Shiryaev SA, He S (2018). Emetine inhibits Zika and Ebola virus infections through two molecular mechanisms: inhibiting viral replication and decreasing viral entry. Cell Discov.

[175] Li Z, Garner AL, Gloeckner C, Janda KD, Carlow CK (2011). Targeting the *Wolbachia* cell division protein FtsZ as a new approach for antifilarial therapy. PLoS Negl Trop Dis.

[176] Hellmann JK, Munter S, Wink M, Frischknecht F (2010). Synergistic and additive effects of epigallocatechin gallate and digitonin on *Plasmodium* sporozoite survival and motility. PLoS ONE.

[177] Lopatriello A, Soré H, Habluetzel A, Parapini S, D’Alessandro S, Taramelli D (2019). Identification of a potent and selective gametocytocidal antimalarial agent from the stem barks of *Lophira*
*lanceolata*. Bioorg Chem.

[178] Al-Abd NM, Nor ZM, Junaid QO, Mansor M, Hasan MS, Kassim M (2017). Antifilarial activity of caffeic acid phenethyl ester on *Brugia*
*pahangi* in vitro and in vivo. Pathog Glob Health.

[179] Lakshmi V, Joseph SK, Srivastava S, Verma SK, Sahoo MK, Dube V (2010). Antifilarial activity in vitro and in vivo of some flavonoids tested against *Brugia*
*malayi*. Acta Trop.

[180] Allard P-M, Dau ETH, Eydoux C, Guillemot J-C, Dumontet V, Poullain C (2011). Alkylated flavanones from the bark of *Cryptocarya*
*chartacea* as dengue virus NS5 polymerase inhibitors. J Nat Prod.

[181] Zandi K, Teoh B-T, Sam S-S, Wong P-F, Mustafa MR, AbuBakar S (2012). Novel antiviral activity of baicalein against dengue virus. BMC Complement Altern Med.

[182] Low ZX, OuYong BM, Hassandarvish P, Poh CL, Ramanathan B (2021). Antiviral activity of silymarin and baicalein against dengue virus. Sci Rep.

[183] Coulerie P, Nour M, Maciuk A, Eydoux C, Guillemot J-C, Lebouvier N (2013). Structure-activity relationship study of biflavonoids on the Dengue virus polymerase DENV-NS5 RdRp. Planta Med.

[184] Gómez-Calderón C, Mesa-Castro C, Robledo S, Gómez S, Bolivar-Avila S, Diaz-Castillo F (2017). Antiviral effect of compounds derived from the seeds of *Mammea*
*americana* and *Tabernaemontana*
*cymosa* on Dengue and Chikungunya virus infections. BMC Complement Altern Med.

[185] Kanyaboon P, Saelee T, Suroengrit A, Hengphasatporn K, Rungrotmongkol T, Chavasiri W (2018). Cardol triene inhibits dengue infectivity by targeting kl loops and preventing envelope fusion. Sci Rep.

[186] Saleem HN, Batool F, Mansoor HJ, Shahzad-ul-Hussan S, Saeed M (2019). Inhibition of dengue virus protease by eugeniin, isobiflorin, and biflorin isolated from the flower buds of *Syzygium*
*aromaticum* (Cloves). ACS Omega.

[187] Weber C, Sliva K, von Rhein C, Kümmerer BM, Schnierle BS (2015). The green tea catechin, epigallocatechin gallate inhibits chikungunya virus infection. Antiviral Res.

[188] Lu J-W, Hsieh P-S, Lin C-C, Hu M-K, Huang S-M, Wang Y-M (2017). Synergistic effects of combination treatment using EGCG and suramin against the chikungunya virus. Biochem Biophys Res Commun.

[189] Henss L, Scholz T, Grünweller A, Schnierle BS (2018). Silvestrol inhibits chikungunya virus replication. Viruses.

[190] Basic M, Elgner F, Bender D, Sabino C, Herrlein M-L, Roth H (2019). A synthetic derivative of houttuynoid B prevents cell entry of Zika virus. Antiviral Res.

[191] Chu JJH, Leong PWH, Ng ML (2006). Analysis of the endocytic pathway mediating the infectious entry of mosquito-borne flavivirus West Nile into *Aedes*
*albopictus* mosquito (C6/36) cells. Virology.

[192] Soré H, Lopatriello A, Ebstie YA, TenohGuedoung AR, Hilou A, Pereira JA (2020). *Plasmodium* stage-selective antimalarials from *Lophira*
*lanceolata* stem bark. Phytochemistry.

[193] Arango E, Londoño B, Segura C, Solarte Y, Herrera S, Saez J (2006). Prevention of sporogony of *Plasmodium* vivax in *Anopheles*
*albimanus* by steroids of *Solanum*
*nudum* Dunal (Solanaceae). Phyther Res.

[194] Ferreira TN, Pita-Pereira D, Costa SG, Brazil RP, Moraes CS, Díaz-Albiter HM (2018). Transmission blocking sugar baits for the control of *Leishmania* development inside sand flies using environmentally friendly beta-glycosides and their aglycones. Parasit Vectors.

[195] Dhananjeyan MR, Milev YP, Kron MA, Nair MG (2005). Synthesis and activity of substituted anthraquinones against a human filarial parasite, *Brugia*
*malayi*. J Med Chem.

[196] Yadav D, Singh SC, Verma RK, Saxena K, Verma R, Murthy PK (2013). Antifilarial diarylheptanoids from *Alnus*
*nepalensis* leaves growing in high altitude areas of Uttarakhand. India Phytomedicine.

[197] Bourjot M, Leyssen P, Eydoux C, Guillemot J-C, Canard B, Rasoanaivo P (2012). Chemical constituents of *Anacolosa*
*pervilleana* and their antiviral activities. Fitoterapia.

[198] da Costa ECB, Amorim R, da Silva FC, Rocha DR, Papa MP, de Arruda LB (2013). Synthetic 1,4-Pyran naphthoquinones are potent inhibitors of dengue virus replication. PLoS ONE.

[199] Cheung YY, Chen KC, Chen H, Seng EK, Chu JJH (2014). Antiviral activity of lanatoside C against dengue virus infection. Antiviral Res.

[200] Guo J, Jia X, Liu Y, Wang S, Cao J, Zhang B (2020). Inhibition of Na+/K+ ATPase blocks Zika virus infection in mice. Commun Biol.

[201] Zanello PR, Koishi AC, de Rezende Júnior CO, Oliveira LA, Pereira AA, de Almeida MV (2015). Quinic acid derivatives inhibit dengue virus replication *in*
*vitro*. Virol J..

[202] Martinez-Lopez A, Persaud M, Chavez MP, Zhang H, Rong L, Liu S (2019). Glycosylated diphyllin as a broad-spectrum antiviral agent against Zika virus. EBioMedicine.

[203] Silva S, Shimizu JF, de Oliveira DM, de Assis LR, Bittar C, Mottin M (2019). A diarylamine derived from anthranilic acid inhibits ZIKV replication. Sci Rep.

[204] Rohrich CR, Ngwa CJ, Wiesner J, Schmidtberg H, Degenkolb T, Kollewe C (2012). Harmonine, a defence compound from the harlequin ladybird, inhibits mycobacterial growth and demonstrates multi-stage antimalarial activity. Biol Lett.

[205] Carter V, Underhill A, Baber I, Sylla L, Baby M, Larget- I (2013). Killer bee molecules: antimicrobial peptides as effector molecules to target sporogonic stages of *Plasmodium*. PLoS Pathog.

[206] Moreira LA, Ito J, Ghosh A, Devenport M, Zieler H, Abraham EG (2002). Bee venom phospholipase inhibits malaria parasite development in transgenic mosquitoes. J Biol Chem.

[207] Moreira CK, Rodrigues FG, Ghosh A, de Varotti FP, Miranda A, Daffre S (2007). Effect of the antimicrobial peptide gomesin against different life stages of *Plasmodium* spp. Exp Parasitol..

[208] O’Neal AJ, Butler LR, Rolandelli A, Gilk SD, Pedra JHF (2020). Lipid hijacking: a unifying theme in vector-borne diseases. Elife.

[209] Werling K, Shaw WR, Itoe MA, Westervelt KA, Marcenac P, Paton DG (2019). Steroid hormone function controls non-competitive *Plasmodium* development in *Anopheles*. Cell.

[210] van Schaijk BC, Santha KTR, Vos WVM, Adam R, van Geert-Jan G, Tao L (2014). Type II fatty acid biosynthesis is essential for *Plasmodium*
*falciparum* sporozoite development in the midgut of *Anopheles* mosquitoes. Eukaryot Cell..

[211] Reynolds RA, Kwon H, Alves E Silva TL, Olivas J, Vega-Rodriguez J, Smith RC (2020). The 20-hydroxyecdysone agonist, halofenozide, promotes anti-*Plasmodium* immunity in *Anopheles*
*gambiae* via the ecdysone receptor. Sci Rep.

[212] Reynolds RA, Hyeogsun K, Smith CR, Photini S (2021). 20-Hydroxyecdysone primes innate immune responses that limit bacterial and malarial parasite survival in *Anopheles*
*gambiae*. mSphere..

[213] Childs LM, Cai FY, Kakani EG, Mitchell SN, Paton D, Gabrieli P (2016). Disrupting mosquito reproduction and parasite development for malaria Control. PLoS Pathog.

[214] Olds CL, Glennon EKK, Luckhart S (2018). Abscisic acid: new perspectives on an ancient universal stress signaling molecule. Microbes Infect.

[215] Nagamune K, Hicks LM, Fux B, Brossier F, Chini EN, Sibley LD (2008). Abscisic acid controls calcium-dependent egress and development in *Toxoplasma*
*gondii*. Nature.

[216] Glennon EKK, Torrevillas BK, Morrissey SF, Ejercito JM, Luckhart S (2017). Abscisic acid induces a transient shift in signaling that enhances NF-κB-mediated parasite killing in the midgut of *Anopheles*
*stephensi* without reducing lifespan or fecundity. Parasit Vectors.

[217] Glennon EKK, Adams LG, Hicks DR, Dehesh K, Luckhart S (2016). Supplementation with abscisic acid reduces malaria disease severity and parasite transmission. Am J Trop Med Hyg.

[218] Glennon EKK, Megawati D, Torrevillas BK, Ssewanyana I, Huang L, Aweeka F (2018). Elevated plasma abscisic acid is associated with asymptomatic *falciparum* malaria and with IgG-/caspase-1-dependent immunity in *Plasmodium*
*yoelii*-infected mice. Sci Rep.

[219] Staveness D, Abdelnabi R, Near KE, Nakagawa Y, Neyts J, Delang L (2016). Inhibition of Chikungunya Virus-induced cell death by salicylate-derived bryostatin analogues provides additional evidence for a PKC-independent pathway. J Nat Prod.

[220] Paton DG, Childs LM, Itoe MA, Holmdahl IE, Buckee CO, Catteruccia F (2019). Exposing *Anopheles* mosquitoes to antimalarials blocks *Plasmodium* parasite transmission. Nature.

[221] Dreyer SM, Leiva D, Magaña M, Pott M, Kay J, Cruz A (2019). Fipronil and ivermectin treatment of cattle reduced the survival and ovarian development of field-collected *Anopheles*
*albimanus* in a pilot trial conducted in northern Belize. Malar J.

[222] Makhanthisa TI, Braack L, Lutermann H (2021). The effect of cattle-administered ivermectin and fipronil on the mortality and fecundity of *Anopheles*
*arabiensis* Patton. Parasit Vectors.

[223] Müller G, Junnila A, Qualls W, Revay EE, Kline DL, Allan S (2010). Control of *Culex*
*quinquefasciatus* in a storm drain system in Florida using attractive toxic sugar baits. Med Vet Entomol.

[224] Junnila A, Revay EE, Müller GC, Kravchenko V, Qualls WA, Xue R (2015). Efficacy of attractive toxic sugar baits (ATSB) against *Aedes*
*albopictus* with garlic oil encapsulated in beta-cyclodextrin as the active ingredient. Acta Trop.

[225] Revay EE, Schlein Y, Tsabari O, Kravchenko V, Qualls W, De-Xue R (2015). Formulation of attractive toxic sugar bait (ATSB) with safe EPA-exempt substance significantly diminishes the *Anopheles*
*sergentii* population in a desert oasis. Acta Trop.

[226] Traore MM, Junnila A, Traore SF, Doumbia S, Revay EE, Kravchenko VD (2020). Large-scale field trial of attractive toxic sugar baits (ATSB) for the control of malaria vector mosquitoes in Mali. West Africa Malar J.

[227] Tenywa FC, Kambagha A, Saddler A, Maia MF (2017). The development of an ivermectin-based attractive toxic sugar bait (ATSB) to target *Anopheles*
*arabiensis*. Malar J.

[228] Andriessen R, Snetselaar J, Suer RA, Osinga AJ, Deschietere J, Lyimo IN (2015). Electrostatic coating enhances bioavailability of insecticides and breaks pyrethroid resistance in mosquitoes. Proc Natl Acad Sci USA.

[229] Mathias DK, Pastrana-mena R, Ranucci E, Tao D, Ferruti P, Ortega C (2013). A small molecule glycosaminoglycan mimetic blocks *Plasmodium* invasion of the mosquito midgut. PLoS Pathog.

[230] Lantero E, Fernandes J, Aláez-Versón CR, Gomes J, Silveira H, Nogueira F (2020). Heparin administered to *Anopheles* in membrane feeding assays blocks *Plasmodium* development in the mosquito. Biomolecules.

[231] Mongkol W, Arunyawat U, Surat W, Kubera A (2015). Active compounds against *Anopheles*
*minimus* carboxypeptidase B for malaria transmission-blocking strategy. J Med Entomol.

[232] Urbán P, Ranucci E, Fernàndez-busquets X (2015). Polyamidoamine nanoparticles as nanocarriers for the drug delivery to malaria parasite stages in the mosquito vector. Nanomedicine.

[233] Cubillos-Ruiz A, Guo T, Sokolovska A, Miller PF, Collins JJ, Lu TK (2021). Engineering living therapeutics with synthetic biology. Nat Rev Drug Discov.

