# Supplementary material for: Contemporary exploitation of natural products for arthropod-borne pathogen transmission-blocking interventions
Source: Parasit Vectors. 2022 Aug 24;15:298. doi: 10.1186/s13071-022-05367-8 (PMC9404607; doi:10.1186/s13071-022-05367-8)
Supplement: Supplementary file 1 — Additional file 1. Summarized details of the highlighted compounds 1 - 151: their chemical names, class, pathogens tested, and described mode of action. [file 13071_2022_5367_MOESM1_ESM.pdf]

Supplementary Table 1

**Compound name**	**Chemical structure number**	**Chemical class**	**Source**	**Vector-borne pathogen(s) tested**	**Described mode/mechanism(s) of action**	**Reference(s)**
	**Microbial derived compounds**					
Romidepsin	**1**	Depsipeptide	*Chromobacterium* spp.	*Plasmodium falciparum* sporogonic stages	Histone deacetylase inhibition	[34]
Violacein	**2**	Bisindole	*Chromobacterium violaceum*	*P. falciparum* gametocytes, ookinetes	Proteostasis collapse	[37]
Epoxomicin	**3**	Morpholine tripeptide	Actinomycete strain Q996-17	*P. falciparum* gametocytes	Proteosome inhibition	[46]
Thiostrepton	**4**	Thiopeptide	*Streptomycetes* spp.	*P. falciparum* gametocytes	Apicoplast and proteosome inhibition	[47]
Ivermectin	**5**	Macrocyclic lactone	*Streptomyces avermectinius*	*P. falciparum* sporogonic stages, ZIKV, WNV, DENV, CHKV	ND for *P. falciparum*; Interaction with viral helicase protein 3	[49-52; 88]
Chlorotonil A	**6**	Tricyclic macrolide	*Sorangium*	*P. falciparum*	ND	[58]

			cellulosum	gametocytes		
BT37	**7**	Dibenzofurandione	(+)-Usnic acid derivative	*P. falciparum* gametocytes, ookinetes	Inner membrane complex 1 (IMC1) and NEK2/4 inhibition	[59]
BT122	**8**	Dibenzofurandione	(+)-Usnic acid derivative	*P. falciparum* gametocytes, ookinetes	Inner membrane complex 1 (IMC1) and NEK2/4 inhibition	[59]
p-orlandin	**9**	Bicoumarin	*Aspergillus niger*	*P. falciparum* gametocytes	FREP1 inhibition	[62]
Asperaculane B	**10**	Sesquiterpenoid	*Aspergillus aculeatus*	*P. falciparum* gametocytes	FREP1 inhibition	[63]
Pulixin	**11**	Benzo[c]chromenone (Coumarin)	*Purpureocillium lilacinum*	*P. falciparum* gametocytes	FREP1 inhibition	[64]
Monensin A	**12**	Polyether ionophore	*Streptomyces* spp	*P. falciparum* gametocytes	Ionic balance disruption	[68]
Nigericin	**13**	Polyether ionophore	*Streptomyces* spp	*P. falciparum* gametocytes	Ionic balance disruption	[68]
Salinomycin	**14**	Polyether ionophore	*Streptomyces* spp	*P. falciparum* gametocytes	Ionic balance disruption	[68]
Narasin	**15**	Polyether ionophore	*Streptomyces* spp	*P. falciparum* gametocytes	Ionic balance disruption	[66]
Maduramicin	**16**	Polyether ionophore	*Streptomyces* spp	*P. falciparum*	Ionic balance disruption	[66, 67]

				gametocytes		
NITD609	**17**	Spiroindolone		*P. falciparum* gametocytes	PfATP4 inhibition	[70, 71]
Corallopyronin A (CorA)	**18**	Polyketide-derived α-pyrone antibiotic	*Corallococcus coralloides* B035	Filarial worms (*Litomosoides sigmodontis*)	Bacterial RNA polymerase inhibition and *Wolbachia* depletion	[72, 73]
Kirromycin B	**19**	Elfamycin antibiotic	*Streptomyces* sp. CB00686	Filarial worms (*Brugia pahangi*)	EF-Tu activity inhibition and *Wolbachia* depletion	[74]
Kirromycin	**20**	Elfamycin antibiotic	*Streptomyces* sp. CB00686	Filarial worms (*Brugia pahangi*)	EF-Tu activity inhibition and *Wolbachia* depletion	[74]
Kirromycin C	**21**	Elfamycin antibiotic	*Streptomyces* sp. CB00686	Filarial worms (*Brugia pahangi*)	EF-Tu activity inhibition and *Wolbachia* depletion	[74]
ABBV-4083	**22**	Macrolide	tylosin A derivative	Filarial worms (*Litomosoides sigmodontis*)	*Wolbachia* depletion	[75]
AN11251	**23**	boron-pleuromutilin		Filarial worms	*Wolbachia* depletion	[76]

				(*Litomosoides sigmodontis*)		
Globomycin	**24**	Cyclic lipopeptide antibiotic	*Streptomyces* spp.	*Brugia malayi*	Lipoprotein biosynthesis inhibition and *Wolbachia* depletion	[77]
Doxycycline	**25**	Tetracycline antibiotic	Synthetic derivative of tetracycline	*Brugia* sp.; CHIKV, ZIKV	*Wolbachia* depletion; ND for the arboviruses	[79; 97]
Minocycline	**26**	Tetracycline antibiotic	Synthetic derivative of tetracycline	*Brugia* sp.	*Wolbachia* depletion	[79]
Rifampicin	**27**	Polyketide	*Amycolatopsis rifamycinica*	*Brugia* sp.	*Wolbachia* depletion	[79]
Azithromycin	**28**	Macrolide	Semi-synthetic derivative of erythromycin	*Brugia* sp.; CHIKV, ZIKV	*Wolbachia* depletion; ND for arboviruses	[79; 96]
Tirandamycin B	**29**	Tetramic acid antibiotic	*Streptomyces* sp. 17944	*B. malayi*	Inhibition of asparaginyl-tRNA synthetase activity	[83]
WS9326A	**30**	Cyclodepsipetide	*Streptomyces violaceoniger* 9078	*B. malayi*	Inhibition of asparaginyl-tRNA synthetase activity	[82]
Adipostatin compound	**31**	Alkylresorcinol	*Streptomyces* sp. 4875	*B. malayi*	Inhibition of asparaginyl-tRNA synthetase activity	[81]

Nanchangmycin	**32**	Polyether ionophore antibiotic	*Streptomyces nanchangensis*	ZIKV, CHIKV, DENV, WNV	Inhibition of viral cell entry	[84]
Cavinafungin	**33**	Lipopeptide	*Colispora cavincola*	ZIKV, DENV 1-4	Inhibition of host and viral protein processing	[85]
Soraphen A	**34**	Polyketide		DENV	Host cell acetyl-CoA carboxylase inhibition	[86]
Labyrinthopeptins A1	**35**	Lantibiotic peptide	*Actinomadura namibiensis*	DENV, CHIKV, WNV, ZIKV	Binding to phosphatidylethanolamine on viral membranes	[87]
Labyrinthopeptins A2	**36**	Lantibiotic peptide	*Actinomadura namibiensis*	DENV, CHIKV, WNV, ZIKV	Binding to phosphatidylethanolamine on viral membranes	[87]
Mycophenolic acid (MPA)	**37**	2-benzofuran	*Penicillium stoloniferum*	DENV, CHIKV, WNV, ZIKV	Inhibition of host cell inosine-5'-monophosphate (IMPDH)	[88,
Cyclosporine A	**38**	Cyclic peptide	*Trichoderma polysporum*	DENV, ZIKV	Inhibition of viral RNA synthesis	[88, 92]
Daptomycin	**39**	Lipopeptide	*Streptomyces roseosporus*	ZIKV	Inhibition of viral cell entry	[88]
Antimycin A1a	**40**	Macrocyclic lactone	*Streptomyces kaviengensis*	DENV	ND	[94]
Acetylspiramycin	**41**	Macrolide	*Streptomyces*	DENV	ND	[94]

			ambofaciens			
Brefeldin A	**42**	Polyketone	*Penicillium* sp.	DENV	ND	[95]
Abamectin	**43**	Macrolide	*Streptomyces avermitilis*	CHIKV	ND	[90]
Debromoaplysiatoxin	**44**	Aplysiatoxin	*Trichodesmium erythraeum*	CHIKV	ND	[98]
Bafilomycin	**45**	Macrolide	*Streptomyces* spp.	DENV-2	Inhibition of vacuolar H+-ATPase (vATPase)	[99]
	**Plant-derived compounds**					
Azadirachtin A	**46**	Triterpenoid limonoid	Neem (*Azadirachtica indica*)	*P. berghei* gametes	Disruption of mitotic microtubule arrays and axonemes in activated male gametes	[118]
Gedunin	**47**	Limonoid	*Azadirachtica indica; Xylocarpus granatum*	*P. falciparum*, *P. berghei* ookinetes; *B. malayi*	Inhibition of heat shock protein 90 (HSP90); Worm immobilization	[69; 146]
Deacetylnimbin	**48**	Furan limonoid	*Azadirachtica indica*	*P. berghei ookinetes*	ND	[119]
Vernodalol	**49**	Sesquiterpene lactone	*Vernonia amygdalina*	*P. falciparum* gametocytes, *P. berghei*	ND	[122]

				ookinetes		
Daucovirgolide G	**50**	Germacranolide sesquiterpenoid	*Daucus virgatus*	*P. berghei* ookinetes	ND	[125]
6-*O*-angeloxyl-8-*O*-senecioyl-6β,8α,11-trihydroxygermacra-1(10)*E*,4*E*-diene	**51**	Polyoxygenated germacrane	*Daucus carota*	*P. berghei* ookinetes	ND	[123]
Parthenin	**52**	Sesquiterpene lactone	*Parthenium hysterophorous*	*P. falciparum* gametocytes, ookinetes	ND	[126]
Parthenolide	**53**	Sesquiterpene lactone	*Tanacetum parthenium*	*P. falciparum* gametocytes, ookinetes	ND	[126]
1α,4α -dihydroxybishopsolicepolide	**54**	Guaianolide sesquiterpenoid	*Artemisia afra*	*P. falciparum* gametocytes	ND	[127]
Artemisone	**55**	Sesquiterpenoid	Artemisinin derivative	*P. falciparum* gametocytes	Redox imbalance	[131]
Artemiside	**56**	Sesquiterpenoid	Artemisinin derivative	*P. falciparum* gametocytes	Redox imbalance	[131]
10-aminoartemisinin compound	**57**	Sesquiterpenoid	Artemisinin derivative	*P. falciparum* gametocytes	Redox imbalance	[132]
10-aminoartemisinin compound	**58**	Sesquiterpenoid	Artemisinin derivative	*P. falciparum* gametocytes	Redox imbalance	[132]
10-aminoartemisinin	**59**	Sesquiterpenoid	Artemisinin	*P. falciparum*	Redox imbalance	[132]

compound			derivative	gametocytes		
10-aminoartemisinin compound	**60**	Sesquiterpenoid	Artemisinin derivative	*P. falciparum* gametocytes	Redox imbalance	[132]
Trigocherriolide A	**61**	Oxygenated diterpenoid	*Trigonostemon cherrieri*	DENV	Inhibition of NS5 RdRp activity	[133]
Prostratin	**62**	Phorbol ester	*Homalanthus nutans*	CHIKV	ND	[134]
12-*O*-tetradecanoylphorbol 13-acetate	**63**	Phorbol ester	*Homalanthus nutans*	CHIKV	ND	[134]
Trigocherrierin A	**64**	Daphane diterpenoid orthoester	*Trigonostemon cherrieri*	CHIKV	ND	[135]
Trigocherriolide E	**65**	Daphane diterpenoid orthoester	*Trigonostemon cherrieri*	CHIKV	ND	[135]
12-*O*-decanoylphorbol 13-acetate	**66**	Tigliane diterpene	*Croton mauritianus*	CHIKV	ND	[136]
12-*O*-decanoyl-7-hydroperoxy-phorbol-5-ene-13-acetate	**67**	Tigliane diterpene	*Croton mauritianus*	CHIKV	ND	[136]
(*2R,3R,4S,5R,7S,8R,13R,15R*)-3,5,7,15-tetraacetoxy-2-hydroxy-8-tigloyloxy-9,14-dioxojatropha-6(17),11*E*-diene	**68**	Acetoxylated jatrophane diterpene	*Euphorbia amygdaloides*	CHIKV	ND	[137]
Phorbol-12,13-didecanoate	**69**	Diterpenoid		CHIKV	ND	[138]

Tonantzitlolone B	**70**	Tonantzitlolone-type diterpene	*Stillingia lineata*	CHIKV	ND	[139]
12-deoxyphorbol-13(2"-methyl)butyrate	**71**	Tigliane diterpenoid	*Stillingia lineata*	CHIKV	ND	[140]
Stachyonic acid A	**72**	Labdane diterpene	*Basilicum polystachyon*	DENV	ND	[141]
Compound 73	**73**	Triterpene glycoside	Glycyrrhizic acid derivative	DENV	ND	[142]
Compound 74	**74**	Triterpene glycoside	Glycyrrhizic acid derivative	DENV	ND	[142]
Compound 75	**75**	Pentacyclic triterpenoid	Glycyrrhetinic acid derivative	ZIKV	ND	[143]
Compound 76	**76**	Pentacyclic triterpenoid	Glycyrrhetinic acid derivative	ZIKV	ND	[143]
Compound 77	**77**	Pentacyclic triterpenoid	Glycyrrhetinic acid derivative	ZIKV	ND	[143]
Compound 78	**78**	Pentacyclic triterpenoid	Glycyrrhetinic acid derivative	ZIKV	ND	[143]
((*4R,9S,14S*)-4α-acetoxy-9β,14α-dihidroxydolast-1(15),7-diene	**79**	Dolastane diterpene	*Canistrocarpus cervicornis*	ZIKV, CHIKV	ND	[144]
Betulinic acid	**80**	Triterpenoid		ZIKV, DENV	Inhibition of viral RNA replication step	[145]

Photogedunin	**81**	Limonoid	*Xylocarpus granatum*	*B. malayi*	Worm immobilization	[146]
Analog compound 82	**82**	Pentacyclic triterpenoid	Glycyrrhetinic acid amide derivative	*B. malayi*	ND	[147]
Ursolic acid	**83**	Pentacyclic triterpenoid	*Nyctanthes arbortristis*	*Wuchereria bancrofti*	Redox imbalance	[148]
Labda-8(20),13-diene-15-oic acid	**84**	Diterpenoid	*Taxodium distichum*	*B. malayi*	ND	[149]
Quinine	**85**	Quinoline methanol	Cinchona tree	*P. vivax, P. malariae* gametocytes	ND	[152, 153]
Securinine	**86**	Tetracyclic alkaloid	*Securinega suffruticosa*	*P. falciparum* gametocytes	Allosteric binding of 2'-deoxyuridine 5'-triphosphate nucleotidohydrolase (PfdUTPase)	[154]
Virosecurinine	**87**	Tetracyclic alkaloid	Securinine derivative	*P. falciparum* gametocytes	Allosteric binding of 2'-deoxyuridine 5'-triphosphate nucleotidohydrolase (PfdUTPase)	[154]
Allosecurinine	**88**	Tetracyclic alkaloid	Securinine derivative	*P. falciparum* gametocytes	Allosteric binding of 2'-deoxyuridine 5'-triphosphate	[154]

					nucleotidohydrolase (PfdUTPase)	
Cryptolepine	**89**	Indoloquinoline	*Cryptolepis sanguinolenta*	*P. falciparum* gametocytes	ND	[155]
3-chloro-8-nitro-tryptanthrin, 3-chloro-8-nitro-indolo [2,1-*b*] quinazoline-6,12-dione (NT1)	**90**	Indoloquinazoline	Tryptanthrin derivative	*P. falciparum* gametocytes	ND	[156]
3-chloro-indolo [2,1-*b*] quinazoline-6,12-dione (T8)	**91**	Indoloquinazoline	Tryptanthrin derivative	*P. falciparum* gametocytes	ND	[156]
Dihydronitidine	**92**	Isoquinoline alkaloid	*Zanthoxylum heitzii*	*P. berghei* ookinetes	ND	[157]
Jozimine A_2	**93**	Naphthylisoquinoline alkaloid	*Ancistrocladus* spp.	*P. falciparum* gametocytes	ND	[158]
Dioncophylline C	**94**	Naphthylisoquinoline alkaloid	*Triphyophyllum peltatum*	*P. falciparum* gametocytes	ND	[158]
Ealapasamine C	**95**	Naphthylisoquinoline alkaloid	*Ancistrocladus ealaensis*	*P. falciparum* gametocytes	ND	[158]
Dimer compound (**96**)	**96**	Naphthylisoquinoline alkaloid		*P. falciparum* gametocytes	ND	[158]
Compound 97	**97**	Naphthylisoquinoline alkaloid		*P. falciparum* gametocytes	ND	[158]
(-)-*R*,*S*-dehydroemetine	**98**	Pyridoisoquinoline	Emetine synthetic derivative	*P. falciparum* gametocytes	ND	[159]

Juliprosopine	**99**	Indolizidine alkaloid	*Prosopis juliflora*	*P. falciparum* gametocytes, ookinetes	ND	[160]
Tazopsine	**100**	Morphinan alkaloid	*Strychnopsis thouarsii*	*P. yoelii*, *P. falciparum* liver stages	ND	[161]
NCP-tazopsine	**101**	Morphinan alkaloid	Tazopsine derivative	*P. yoelii*, *P. falciparum* liver stages	ND	[161]
Sinococuline	**102**	Morphinan alkaloid	*S. thouarsii*	*P. yoelii* liver stages	ND	[161]
Berberine	**103**	Isoquinoline		CHIKV; *B. malayi*	Mitogen-activated protein kinase (MAPK) signalling and viral nucleocapsid assembly inhibition; inhibition of *Wolbachia* FtsZ GTPase activity	[90, 164, 165; 176]
Harringtonine	**104**	Cephalotaxine ester	*Cephalotaxus harringtonia*	CHIKV	Inhibition of viral protein synthesis	[166]
Halofuginone	**105**	Quinazolinone alkaloid	Febrifugine derivative	DENV, CHIKV	Targeting host translational machinery	[167]
Tomatidine	**106**	Steroidal alkaloid	Unripe tomatoes (*Solanum*	CHIKV, DENV	ND	[168, 169]

			lycopersicum)			
Castanospermine	**107**	Indolizidine alkaloid	*Castanospermum australe*	DENV 1-4	Inhibition of host cell α-glucosidase activity	[170]
Lycorine	**108**	Indolizidine alkaloid	Amaryllidaceae plant family	DENV, YFV, ZIKV, WNV, RVFV	Inhibition of viral RNA replication and protein synthesis	[171-173]
1-acetyllycorine analog	**109**	Indolizidine alkaloid	Lycorine derivative	DENV, YFV, ZIKV, WNV, RVFV	Inhibition of viral RNA replication and protein synthesis	[171]
Cherylline	**110**	Isoquinoline alkaloid	*Crinum jagus*	DENV, ZIKV	Interfering with viral RNA synthesis post-entry step	[174]
Emetine	**111**	Pyridoisoquinoline alkaloid	*Psychotria ipecacuanha*	ZIKV	Inhibition of ZIKV NS5 polymerase activity and disrupting lysosomal function	[175]
Epigallocatechin gallate	**112**	Polyphenol	*Camellia sinensis*	*P. falciparum* sporozoites; CHIKV	Impairment of sporozoite gliding motility; Viral cell entry inhibition	[177; 188, 189]
Lophirone E	**113**	Biflavonoid	*Lophira lanceolata*	*P. falciparum* gametocytes	ND	[178]
Caffeic acid phenethyl ester	**114**	Alkyl caffeate ester (phenolic compound)	*Melaleuca cajuputi*	*B. pahangi*	Depletion of Wolbachia and reduction of FtsZ copy number	[179]

Naringenin	**115**	Flavanone		*B. malayi*	Worm immobilization	[180]
Chartaceone C	**116**	Dialkylated flavanone	*Cryptocarya chartacea*	DENV-2	Inhibition of NS5 RNA-dependent RNA polymerase (RdRp) activity	[181]
Chartaceone D	**117**	Dialkylated flavanone	*Cryptocarya chartacea*	DENV-2	Inhibition of NS5 RNA-dependent RNA polymerase (RdRp) activity	[181]
Chartaceone E	**118**	Dialkylated flavanone	*Cryptocarya chartacea*	DENV-2	Inhibition of NS5 RNA-dependent RNA polymerase (RdRp) activity	[181]
Chartaceone F	**119**	Dialkylated flavanone	*Cryptocarya chartacea*	DENV-2	Inhibition of NS5 RNA-dependent RNA polymerase (RdRp) activity	[181]
Baicalein	**120**	Flavonoid	*Scutellaria baicalensis*	DENV-2	Blocking viral attachment and cell entry	[182, 183]
Sotetsflavone	**121**	7"-*O*-methylamentoflavone	*Dacrydium araucarioides*	DENV	Inhibition of NS5 RdRp activity	[184]
Coumarin A 34SK001	**122**	Coumarin	*Mammea americana*	DENV-2/NG and CHKV-ACol	Inhibition of viral genome replication	[185]

Coumarin B 34SK002	**123**	Coumarin	*Mammea americana*	DENV-2/NG and CHKV-ACol	Inhibition of viral genome replication	[185]
Cardol triene	**124**	Phenolic lipid	Cashew nut shell	DENV	Targeting envelope protein kl loops preventing fusion and infectivity	[186]
Eugeniin	**125**	Ellagitannin	*Syzygium aromaticum*	DENV-2, -3	Inhibition of NS2BNS3pro complex	[187]
Silvestrol	**126**	Cyclopenta[*b*]benzofuran	*Aglaia foveolata*	CHIKV	Inhibition of a host cell DEAD-box helicase eIF4A	[190]
Houttuynoid B derivative (TK1023)	**127**	Flavonoid glycoside	*Houttuynia cordata*	ZIKV	Inhibition of viral cell entry	[191]
Genistein	**128**	Isoflavone		WNV	Disruption of focal adhesion kinase (FAK) functionality at viral entry step	[192]
Lanceolin B	**129**	Lanceolins	*Lophira lanceolata*	*P. falciparum* ookinetes	ND	[193]
SN-2	**130**	Steroids	*Solanum nudum*	*P. vivax* sporogonic stages	ND	[194]
Mandelonitrile	**131**	Aglycone		*Leishmania*	Inhibition of β-	[195]

				spp.	glucosidases	
Esculetin	**132**	Umbelliferone	Esculin hydrolysis	*Leishmania* spp.	Inhibition of β-glucosidases	[195]
Anthraquinone K	**133**	Quinone	*Hemerocallis fulva*	*B. malayi*	Distortions on intrauterine embryos	[196]
Alnus dimer	**134**	Diarylheptanoid	*Alnus nepalensis*	*B. malayi*	ND	[197]
(*5S*)-5- hydroxy-1-(4-hydroxyphenyl)-7-(3,4-dihydroxyphenyl)-3-heptanone	**135**	Diarylheptanoid	*Alnus nepalensis*	*B. malayi*	ND	[197]
Octadeca-9,11,13-triynoic acid	**136**	Acetylenic acid	*Anacolosa pervilleana*	DENV	Inhibition of DENV RdRp activity	[198]
(*13E*)-octadec-13-en-9,11-diynoic acid	**137**	Acetylenic acid	*Anacolosa pervilleana*	DENV	Inhibition of DENV RdRp activity	[198]
(*13E*)-octadec-13-en-11-ynoic acid	**138**	Acetylenic acid	*Anacolosa pervilleana*	DENV	Inhibition of DENV RdRp activity	[198]
Diastereoisomers compound 139	**139**	1,4-pyran naphthoquinone	Lapachol derivative	DENV-2	ND	[199]
Diastereoisomers compound 140	**140**	1,4-pyran naphthoquinone	Lapachol derivative	DENV-2	ND	[199]
Lanatoside C	**141**	Cardiac glycoside	*Digitalis lanata*	DENV	Inhibition of viral RNA and protein synthesis	[200]

Ouabain	**142**	Cardiac glycoside	African ouabio tree	ZIKV	Block viral RNA synthesis by targeting Na^+/K^+-ATPase	[201]
Quinic acid amide derivative	**143**	Cyclitol	Quinic acid derivative	DENV 1-4	ND	[202]
Quinic acid amide derivative	**144**	Cyclitol	Quinic acid derivative	DENV 1-4	ND	[202]
Diphyllin	**145**	Naphthalene-derived compound	*Justicia gendarussa*	ZIKV, WNV, DENV	Prevention of endosomal acidification	[203]
FAM E3	**146**	Aminobenzoic acid	Anthranilic acid diarylamine synthetic derivative	ZIKV	Binding to and stabilizing NS3 helicase	[204]
	**Miscellaneous compounds**					
(17R,9Z)-1,17-diaminooctadec-9-ene (harmonine)	**147**	Insect defense alkaloid	*Harmonia axyridis* (Asian lady bird)	*P. falciparum* NF54 gametocytes, sporogonic stages	ND	[205]
Melittin	**148**	Peptide toxin	Bees (*Apis mellifera*)	*Plasmodium* sporogonic stages	ND	[206]
Ecdysteroid (20E)	**149**	Insect steroid hormone	Mosquito	*P. falciparum*	Non-competitive lipid	[210]

				sporogonic stages	mobilization and boosting of basal innate defenses	
Abscisic acid	**150**	Plant isoprenoid hormone	Plants, human stress signalling molecule	*P. falciparum, P. berghei* gametocytes and sporogonic stages	Priming innate immune activations	[217, 218]
Compound 151	**151**	Macrolide	Bryostatin 1 derivative	CHIKV	PKC-independent mechanisms	[220]

ND: Not defined; DENV: Dengue Fever Virus; YFV: Yellow Fever Virus; ZIKV: Zika Virus; WNV: West Nile Virus; RVFV: Rift Valley Fever Virus; CHIKV: Chikungunya Virus
